# The Role of α‑Synuclein–DNAJB6b Coaggregation in Amyloid Suppression

**DOI:** 10.1021/acschemneuro.4c00883

**Published:** 2025-04-30

**Authors:** Tinna Pálmadóttir, Josef Getachew, Dev Thacker, Johan Wallerstein, Ulf Olsson, Cecilia Emanuelsson, Sara Linse

**Affiliations:** † Biochemistry and Structural Biology, 5193 Lund University, 22100 Lund, Sweden; ‡ Biophysical Chemistry, 90825 Lund University, 22100 Lund, Sweden; § Physical Chemistry, Lund University, 22100 Lund, Sweden

**Keywords:** self-assembly, chaperone action, solubility enhancement, coaggregation, aggregation rate, aggregation equilibrium

## Abstract

Chaperones may retard the aggregation of other proteins and increase their solubility. An important goal is a thermodynamic understanding of such an action. Here, the chaperone DNAJB6b (JB6) is found to suppress amyloid formation of the protein α-synuclein (α-syn) leading to a reduced rate of fibril formation and an increase in apparent solubility of α-syn. These findings were reached at mildly acidic pH and with light seeding under conditions where the effect on secondary nucleation is visible. Cryo-transmission electron microscopy (cryo-TEM) imaging reveals that coaggregates of α-syn and JB6 are formed with significantly altered ultrastructure compared to both pure protein fibrils and pure chaperone aggregates. This is further supported by the formation of ThT-negative aggregates and by the depletion of JB6 from solution in the presence of α-syn. The identification of such coaggregates provides a plausible thermodynamic explanation for an increase in α-syn solubility in the presence of JB6; the reduced chemical potential of the chaperone upon formation of coaggregates can compensate for an increased chemical potential of α-syn, and the system as a whole can lower its free energy to sustain an increased free α-syn concentration.

## Graphical abstract

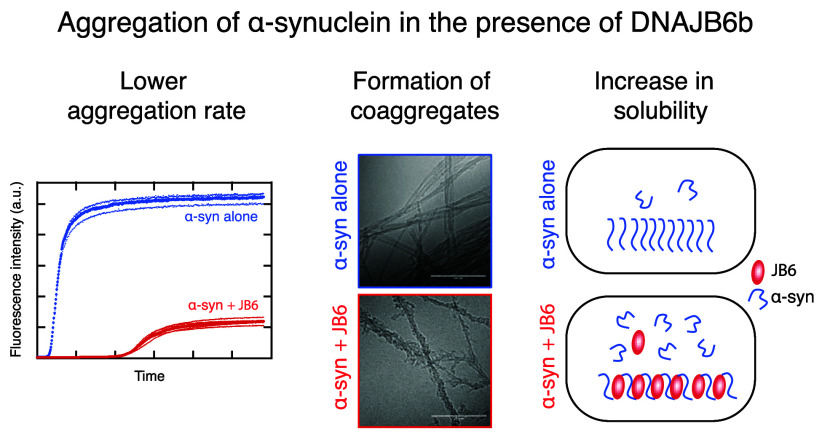

## 1 Introduction

Protein misfolding and aggregation into amyloid fibrils are linked to several neurodegenerative disorders, such as Parkinson’s, Alzheimer’s, and Huntington’s disease.^1–3^ An important group of proteins, named molecular chaperones, are components of the protein quality control system. They may promote folding, prevent misfolding or aggregation of other proteins in vivo and in vitro, and promote degradation through interactions with ubiquitin ligase complexes and proteases as well as through autophagy.^4–8^ Effects are observed both in terms of rate and equilibrium, with the inhibition of aggregation processes starting from excess free monomers and dissociation of excess aggregates.^9–18^ Given the prominent role of chaperones in modulating amyloid formation, comprehensive studies of their impact and the molecular basis of their action may guide the therapeutic interventions of neurodegenerative diseases.

α-syn is found in nearly all neuronal compartments in the human body, and it is enriched in the presynaptic terminals.^19,20^ It consists of 140 residues with a remarkably segregated primary structure that can be divided into three regions: the N-terminal amphipathic region, a central hydrophobic nonamyloid-β component (NAC) region, and the acidic C-terminal tail. It can also be divided into: the N-terminal tail, the fibril core, and the C-terminal tail based on the fold of the monomers in amyloid fibrils (see, for example, 2N0A.pdb) (Figure 1A).^20–23^ In its monomeric form in solution, α-syn, is intrinsically disordered. However, when bound to lipid membranes, its first up to 95 residues may take on an α-helical conformation.^20–22,24^

**Figure 1:**
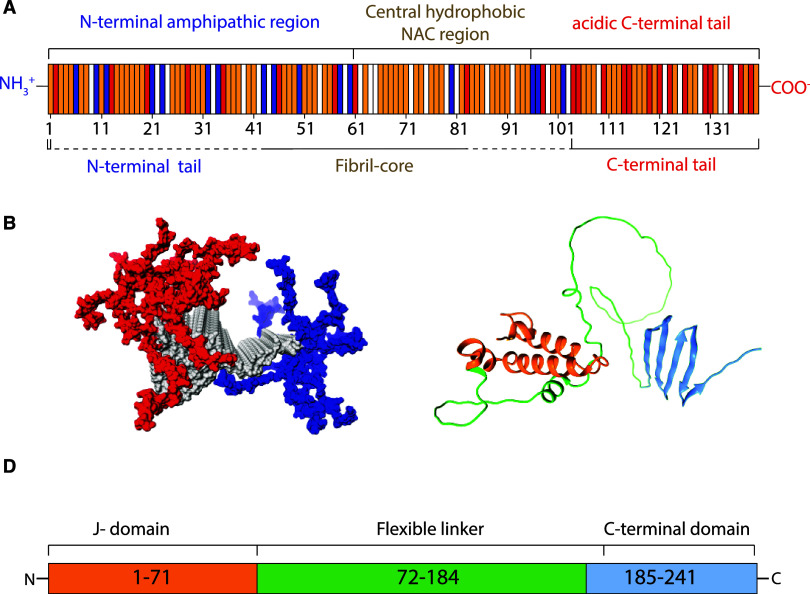
Structures and sequences of α-syn and JB6. (A) Distribution of acidic (red), basic (blue), hydrophobic (yellow), and polar and noncharged (white) residues within the α-syn sequence. At the top of the representation, the 140-residue-long sequence is divided into the N-terminal amphipathic region (1–60), central hydrophobic NAC region (61–95), and C-terminal tail (96–140), based on its primary structure. Below, the representation of the sequence is divided into three regions: N-terminal tail, fibril core, and C-terminal tail based on the folding of the monomers into amyloid fibrils. Comparison between available structural models reveals variation in how many residues belong to the fibril core, ranging from residue 2 to 104 (dashed lines in panel (A)). The C-terminal tail is unstructured in all available structural models. (B) Structure (pdb: 2N0A^58^) of α-syn proto-filaments, showing 10 planes of monomers, colored according to the N-terminal tail (blue), the fibril core (gray), and the C-terminal tail (red) shown as CPK model. (C) Ribbon model of the JB6 monomer predicted by AlphaFold 2 ^52^ colored as J-domain (orange), low-complexity region (green), and CTD (blue). (D) Sequence overview of DNAJB6b.

α-syn has been found to play diverse roles in neuronal physiology and synaptic plasticity, facilitating vesicle trafficking and secretion in cells, such as exo- and endocytosis of synaptic vesicles.^20,25,26^ α-syn can, however, also misfold and form toxic oligomeric species and amyloid fibrils, which can accumulate into Lewy bodies associated with synucleinopathies, such as Parkinson’s disease.^20,27,28^ The fibril formation of α-syn occurs via primary nucleation, secondary nucleation, and elongation under quiescent conditions. To our knowledge, primary nucleation of α-syn in bulk has never been confirmed at physiologically relevant conditions, while heterogeneous nucleation at surfaces is well established.^29–32^ The rate of fibril formation has previously been found to be significantly higher at mildly acidic pH compared to neutral pH, with the rate of secondary nucleation being 4 orders of magnitude higher at pH 5.2 than 7.4.^33–35^ The mildly acidic conditions within late endosomes and secretory granules involved in endo- and exocytosis,^36–38^ motivates studies in this pH range.

DNAJB6b (JB6) belongs to class B of the family of DNAJ molecular chaperones (previously called Hsp40) with over 40 members that may regulate and stabilize the interactions between Hsp70s and their substrates by stimulating the ATPase activity of the Hsp70s.^10,39–41^ JB6 exists within various organs of the human body, including the neuronal cells in the brain.^42,43^ It is present within both the cytosol and the nucleus. JB6 has been shown to suppress Parkinson’s pathology and cell death in mouse and cell models.^39,44,45^ It is present within the center of Lewy bodies and has been found to be downregulated in Parkinson’s disease patients.^39,40,46^ A mutated form of JB6 (T193A) has also been found to increase the risk of Parkinson’s disease in carriers of a mutation in the β-glucocerebrosidase gene.^47^ In vitro, JB6 suppresses the fibril formation of several amyloidogenic proteins such as polyglutamine peptide,^10,13,16^ amyloid β peptide (Aβ),^14,15,48^ and TDP43.^49^

JB6 consists of an α-helical N-terminal domain (residues 1–71, the J-domain), linked to the β-sheet-rich C-terminal domain (residues 185–241, CTD) through a long and flexible linker (residues 72–184).^13,15,50^ The domain structures are reported based on NMR spectroscopic analysis for a variant with a shortened linker.^51^ In an AlphaFold 2 model^52^ of monomeric JB6 shown in Figure 1C, the domains are well predicted, but the flexible linker is not. The linker sequence is of low complexity, abundant in phenylalanine (F) and glycine (G) residues, and a region rich in serine (S) and threonine (T) residues is located within the linker and the CTD (residues 155–195).^15,53^

JB6 self-assembles into large polydisperse oligomers at concentrations above its critical micelle concentration (120 nM).^50^ A recent study indicates that the active form responsible for the retardation of the aggregation of Aβ42 is the small subunit, rather than the larger oligomeric species.^54^ The influence of various molecular chaperones on the aggregation rates and mechanisms of amyloid fibril formation has been thoroughly investigated. Molecular chaperones have been found to interact with the monomers, oligomers, or fibrils and can impact any of the microscopic processes of amyloid fibril formation in a system-dependent manner.^48,55–57^ While intact JB6 inhibits both nucleation steps of Aβ, the isolated CTD inhibits only secondary nucleation in Aβ fibril formation.^14,17^

In addition, molecular chaperones can affect the relative stability of the monomers and fibrillar forms, thus affecting the solubility of the amyloidogenic proteins. For example, JB6 retards the aggregation of Aβ and affects the end state, resulting in higher Aβ solubility in the presence of the chaperone.^9,14,15^

Here we have studied the effect of the chaperone JB6 on the rate of aggregation, as well as the solubility of the amyloidogenic protein α-syn in vitro. The study was performed at mildly acidic pH under light seeding conditions using fluorescence and NMR spectroscopy, chromatography, and electrophoresis, whereby the effects of JB6 on secondary nucleation and solubility can be studied. We also used cryo-transmission electron microscopy (cryo-TEM) to compare the characteristics of the α-syn aggregates formed in the presence and absence of JB6, in addition to quantification of the solution composition. This enables the discussion of a proposed thermodynamic explanation of the observed increase in solubility in the presence of JB6.^9^

## 2 Experimental Section

### 2.1 Expression of α-syn

The gene coding for wild-type human α-syn with Escherichia coli (E. coli)-optimized codons was cloned into a Pet3a-plasmid (purchased from GenScript, Piscataway, New Jersey) with an ATG start codon (corresponding to Met1) (see Section S1). The protein was expressed in E. coli BL21 DE3_pLysS* in LB medium, or autoinduction medium^59^ for unlabeled protein, and in M9 minimal medium with ^15^NHCl_4_ for ^15^N-labeled samples, all containing 30 μg/mL chloramphenicol and 50 μg/mL ampicillin at 37 °C in baffled flasks with 125 rpm shaking. Cells were harvested by centrifugation at 6000*g* for 12 min at 4 °C (JA 8.100 rotor, Avanti J-26 XP centrifuge, Beckman Coulter). The cells obtained from a total of 4 L of culture were combined and mixed with 25 mL of water and stored at −20 °C until further purification. Samples of 1 mL were taken from the culture right before harvesting to test whether the expression was successful (see Section S2). The 1 mL samples were centrifuged at ∼20,000*g*, the supernatant was disposed, and the cell pellets were frozen at −20 °C. The cell pellet was thawed and fully resuspended in 100 μL sterile H_2_O, boiled for 1 min, and centrifuged for 10 min at ∼20,000*g*. The supernatant and the pellet (8 μL) were run on sodium dodecyl sulfate-polyacrylamide gel electrophoresis (SDS-PAGE) using 4–20% precast Tris/Tricine gels (see Figure S1).

### 2.2 Purification of α-syn

The frozen cell pellet from a 4 L culture was resuspended in cold ∼100 mL of buffer A (10 mM Tris/HCl, 1 mM ethylenediaminetetraacetic acid (EDTA), pH 7.5) and placed on ice (volume of buffer versus cell pellet was ca. 5:1). The sample was sonicated using pulsed sonication (1 s on, 1 s off) until the sample was homogeneous. This was done stepwise, and the sample was placed on ice the whole time in order not to heat the sample. After sonication, the sample was centrifuged for 10 min at 15,000*g* at 4 °C (JA 25.50 rotor, Avanti J-26 XP centrifuge, Beckman Coulter). Supernatant was collected and poured into an equal amount of boiling buffer A. The sample was stirred continuously, and the temperature was monitored carefully. When the temperature had reached 85 °C, the sample was placed back on ice, and stirring was continued until the sample had cooled down. If a light-colored pellet remained after the first sonication and centrifugation, the procedure was repeated to maximize the yield. The cooled sample was centrifuged for 10 min at 15,000*g* and 4 °C (JA25.50 rotor, Avanti J-26 XP centrifuge, Beckman Coulter) in order to remove the majority of the precipitated E. coli proteins. The supernatant was collected and saved for further purification.

The first ion-exchange chromatography step was performed using a 3.5 cm diameter column with 100 g diethylaminoethyl (DEAE) cellulose conditioned in buffer A. All buffers were degassed and filtered using a hydrophilic polypropylene membrane filter, 0.2 μm (Pall Corporation), kept cold, and the purification was performed in a cold room. Using a pump, the sample was slowly loaded onto the column (∼2 mL/min), which was then washed with a minimum of 100 mL of buffer A at 1 mL/min and eluted with 1.4 L of linear 0–0.5 M NaCl gradient in buffer A at 1 mL/min. The eluate was collected into fractions and analyzed with agarose gel electrophoresis or SDS-PAGE (Section S3 and Figure S2). The fractions containing mainly α-syn and minimal impurities were combined and diluted in an equal amount of buffer A and further purified by a second ion-exchange chromatography step.

The second ion-exchange chromatography step was performed in the same way as described above, except for this time using 60 g of DEAE sephacel resin and a column of 2.3 cm diameter (see Section S3). The fractions were analyzed by measuring the absorbance at 280 nm. To determine the purity and the presence of α-syn, the fractions showing absorbance at 280 nm were analyzed by SDS-PAGE before the corresponding fractions containing α-syn without any detectable impurities were collected, combined, and stored at −20 °C in 1 mL aliquots. The concentration of the pooled sample was between 70 and 210 μM for all batches based on absorbance at 280 nm, using an extinction coefficient: ε_280nm_ = 5800 M^–1^ cm^–1^. The purity of the sample was confirmed by the absence of any additional bands on SDS-PAGE (Section S3 and Figure S3). The identity and quality were confirmed with mass spectrometry and by performing aggregation kinetics for each batch.

### 2.3 Expression and Purification of DNAJB6

A tag-free DNAJB6b (the gene sequence is presented in Section S4) was expressed in autoinduction medium^59^ and purified using sonication at pH 6.0 and 8.0, removal of contaminants on ion-exchange resins, followed by ammonium sulfate precipitation and two size exclusion steps with and without 1.6 M GuHCl as described.^60^ An SDS-PAGE analysis of the different JB6 batches used in this study is shown in Section S5 and Figure S4.

### 2.4 Monomer Preparation Using Size Exclusion Chromatography

The 1 mL aliquots of α-syn were lyophilized and dissolved in 1.1 mL of 6 M guanidinium hydrochloride and thoroughly mixed using a vortex mixer. The number of tubes dissolved in a total volume of 1.1 mL depends on the required sample concentration. The sample was left at room temperature (RT) for at least an hour to ensure that it completely dissolved. Thereafter, the sample was loaded onto a Superdex 75 Increase 10/300 GL (GE Healthcare) size exclusion column (SEC) using a fast protein liquid chromatography (FPLC) system (Bio-RAD BIOLOgic Fuo Flow, Hercules, CA), which had been equilibrated with the experimental buffer. Buffers were freshly prepared, filtered, and degassed prior to each experiment. The sample was eluted at a flow rate of 0.7 mL/min, and the absorbance at 280 nm was recorded to follow the elution of the monomers. The fractions corresponding to the center of the monomer peak (ca. 1–1.5 mL) (see Section S6 and Figure S5A) were collected into low-binding tubes (Genuine Axygen Quality or Eppendorf Protein LoBind tubes) and kept on ice until the start of the experiment. The concentration of the sample was determined from the absorbance at 280 nm using ε_280nm_ = 5800 M^–1^ cm^–1^. Between monomer isolation rounds, the column was cleaned with 10 mL of 50% formic acid, 10 mL of 0.5 M NaOH, and 10 mL of 1 M Tris, with one column volume of water between each solution. The cleaning steps are critical for maintaining the purity of the samples. The column is stored in 20% EtOH.

### 2.5 Aggregation Kinetics

Aggregation kinetics of α-syn in the presence and absence of JB6 were monitored by measuring thioflavin T (ThT) fluorescence using excitation and emission wavelengths of 440 and 480 nm, respectively. ThT binds to amyloid fibrils, constraining the rotation of a carbon–carbon bond between two rings, leading to an increase in fluorescence quantum yield. 100 μL samples were incubated in a 96-well half-area low-binding polyethylene glycol (PEG)-coated polystyrene plate (3881, Corning) with a transparent bottom and sealed with a SealPlate film (Excel Scientific). Samples were incubated at 37 °C without shaking. The final concentration of α-syn was 20 μM, and the final concentration of JB6 ranged from 7.8 to 125 nM (Figure 2). Experiments were performed in either 10 mM 2-(*N*-morpholino)­ethansulfonic acid (MES), 1.4 mM sodium phosphate (NaP), 0.02% NaN_3_ pH 5.5 or 10 mM acetate, 0.7 mM NaP, 0.02% NaN_3_, pH 4.5. Experiments were performed using 1% of seeds (final concentration 200 nM). The seeds are fibrils formed by incubating 20 μM freshly prepared α-syn in a low-binding tube with 700 rpm stirring using a magnetic stir bar at 37 °C for 2 days (until the final plateau is reached), aliquoted, and frozen at −20 °C. The seeds were made in 10 mM MES/NaOH, pH 5.5, 0.02% NaN_3_ or 10 mM acetate, pH 4.5, 0.02% NaN_3_, depending on the experimental conditions. The free monomer is <0.2 μM in the 20 μM seed stock at pH 5.5, and the free monomer is thus not separated from the seeds before adding them at low concentration (200 nM seed + <2 nM monomer) to the freshly purified 20 μM monomer. Before each aggregation kinetic experiment, the seeds were thawed and placed in a sonication bath for 1 min prior to incubation at RT for a minimum of 1 h.

**Figure 2:**
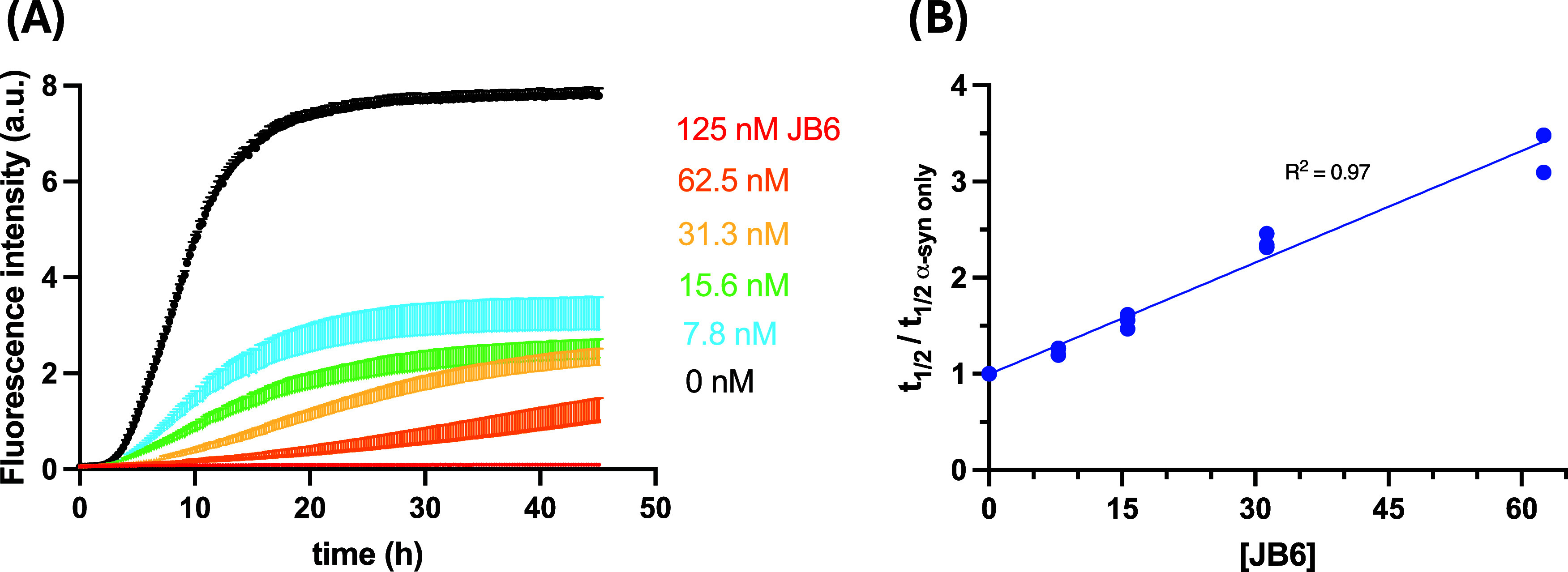
Suppression of α-syn fibril formation by JB6 at a mildly acidic pH at 37 °C. (A) Aggregation was followed by monitoring ThT fluorescence. The aggregation kinetics of 20 μM α-syn at pH 5.5 was studied in the presence of 125 nM (0.6%), 62.5 nM (0.3%), 31.3 nM (0.15%), 15.6 nM (0.08%), and 7.8 nM (0.04%) JB6. Three replicates are shown for each condition. Data for up to 188 h are shown in Section S8. (B) Relative half-times (*t*_1/2_) α-syn in the presence of 62.5 nM (0.3%), 31.3 nM (0.15%), 15.6 nM (0.08%), and 7.8 nM (0.04%) JB6. The half-times positively correlate with JB6 concentration with an *R* ^2^ of 0.97.

### 2.6 Solubility Measurements

#### 2.6.1 Solubility Measured by SDS-PAGE

The kinetic experiments from which samples were collected for solubility determination were performed according to the procedure in Section 2.5 in 10 mM MES/NaOH, 1.4 mM NaP, 0.02% NaN_3_ pH 5.5. Samples were withdrawn from the plate by pipetting up and down a few times and scraping the bottom of the well carefully using the pipet tip. Separation of free monomers and fibrils was done by centrifugation at 31,510*g* for 90 min at RT in 1.5 mL low-binding tubes. The supernatant was analyzed with SDS-PAGE (Section 2.7). From the SDS-PAGE, the intensity of the bands was analyzed by using the software ImageJ.

#### 2.6.2 Solubility Measured by HPLC-MS

The kinetic experiment from which the sample was extracted for solubility determination was performed according to the procedure in Section 2.5 in 10 mM MES, 1.4 mM NaP, and 0.02% NaN_3_, pH 5.5. Seeds used were made in 10 mM MES, 0.02% NaN_3_, and pH 5.5. The α-syn concentrations were 20 μM monomer and 200 nM seeds (1%). The JB6 concentrations ranged from 7.8 to 125 nM. After 45, 140, and 188 h, one of the three replicates at each JB6 concentration was transferred into low-binding tubes and centrifuged at 31,510*g* for a minimum of 1 h. The supernatant was used for quantification by HPLC-MS (Shimadzu Corporation), and the protein concentration was quantified by measuring absorbance during elution of a reversed-phase column (BIOshell A160 Peptide CN, 5 cm × 2.1 mm, 2.7 μM). 10 μL of supernatant was injected onto the column and eluted using a gradient from 5 to 95% acetonitrile in water, with 0.1% trifluoroacetic acid (TFA) at a flow rate of 0.5 mL/min. The absorbance was measured at 205 and 280 nM. Area integration was conducted with the instrument software. A standard curve, recorded for α-syn monomer in the range of 20–0.041 μM, was used. The monomer concentration of the injected supernatant was estimated via the absorbance at either 280 nm (for the higher concentrations) or 205 nm (for the lower concentrations). Additionally, the same samples (supernatants) were analyzed by SDS-PAGE (see Section 2.7).

#### 2.6.3 Solubility Measured by NMR

The samples for the NMR experiments were prepared in 10 mM MES, 1.4 mM NaP, 0.02% NaN_3_, pH 5.5 with 20 μM ^15^N-labeled α-syn, 1% seeds, 10 μM free ^15^N-labeled tryptophan, sodium trimethylsilylpropanesulfonate (DSS), and 10% D_2_O. 550 μL samples were also prepared with or without 250 nM nonlabeled DNAJB6b. One-dimensional (1D) ^1^H and two-dimensional (2D) ^1^H–^15^N heteronuclear single quantum coherence (HSQC) NMR spectra were recorded at 37 °C using a Bruker Avance Neo 800 MHz spectrometer with TCI 5 mm CryoProbe (Bruker Biospin, Rheinstetten, Germany). The 1D ^1^H spectra were recorded using water suppression at 4.7 ppm with excitation sculpting^61^ combined with a perfect echo^62^ using the standard Bruker pulse-program zgesgppe. The spectral width was 15.6 ppm with 32,768 points, 1.3 s acquisition time, 128 scans, and an interscan delay of 1.5 s. The total experimental time was 6 min 30 s. The 2D ^1^H–^15^N HSQC spectra were recorded using sensitivity-enhanced gradient coherence selection^63^ using the standard Bruker pulse-program. The spectral width was 14.2 ppm with 2048 points, 90 ms acquisition time, and transmitter frequency offset at 4.7 ppm in the direct dimension (^1^H). The spectral width in the indirect dimension was 34 ppm, 128 points, and the transmitter frequency offset at 118 ppm. The interscan delay was 1 s, resulting in a total experimental time of 20 min. Data was processed with TopSpin 4.3.0, and an exponential window function with line broadening of 2 Hz was used for the 1D data. The integration of the peaks covered the envelope of the amide region 7.8–9.0 ppm. For processing of the 2D data prior to Fourier transformation, linear prediction and zero filling in the indirect dimension were applied to yield the final data set of 2048 × 512 points.

### 2.7 Sodium Dodecyl Sulfate-Polyacrylamide Gel Electrophoresis (SDS-PAGE) Analysis

Samples were analyzed by SDS-PAGE using Novex 10–20% Tris/Tricine gels (Invitrogen by Thermo Fisher Scientific). Samples were mixed 1:1 with loading buffer, and 10 μL was carefully loaded onto each well. The standard used was a PageRuler prestained protein ladder (Thermo Fisher Scientific, Waltham, MA). Gels were stained using 15 mL InstantBlue Coomassie Protein Stain (ISB1L, Abcam Ltd.).

In addition to the supernatant samples, monomeric α-syn of known concentration was loaded onto the gel (Figures 3 and S9) and used as a concentration reference for the signal intensity of the supernatants. The electrophoresis was run at 70 V for 15 min and at 120 V for 60 min. The gel was washed with Milli-Q water and incubated in the stain with slow rocking at RT overnight. The following day, the staining was replaced with water and left on the shaker for a minimum of 1 h before imaging.

**Figure 3:**
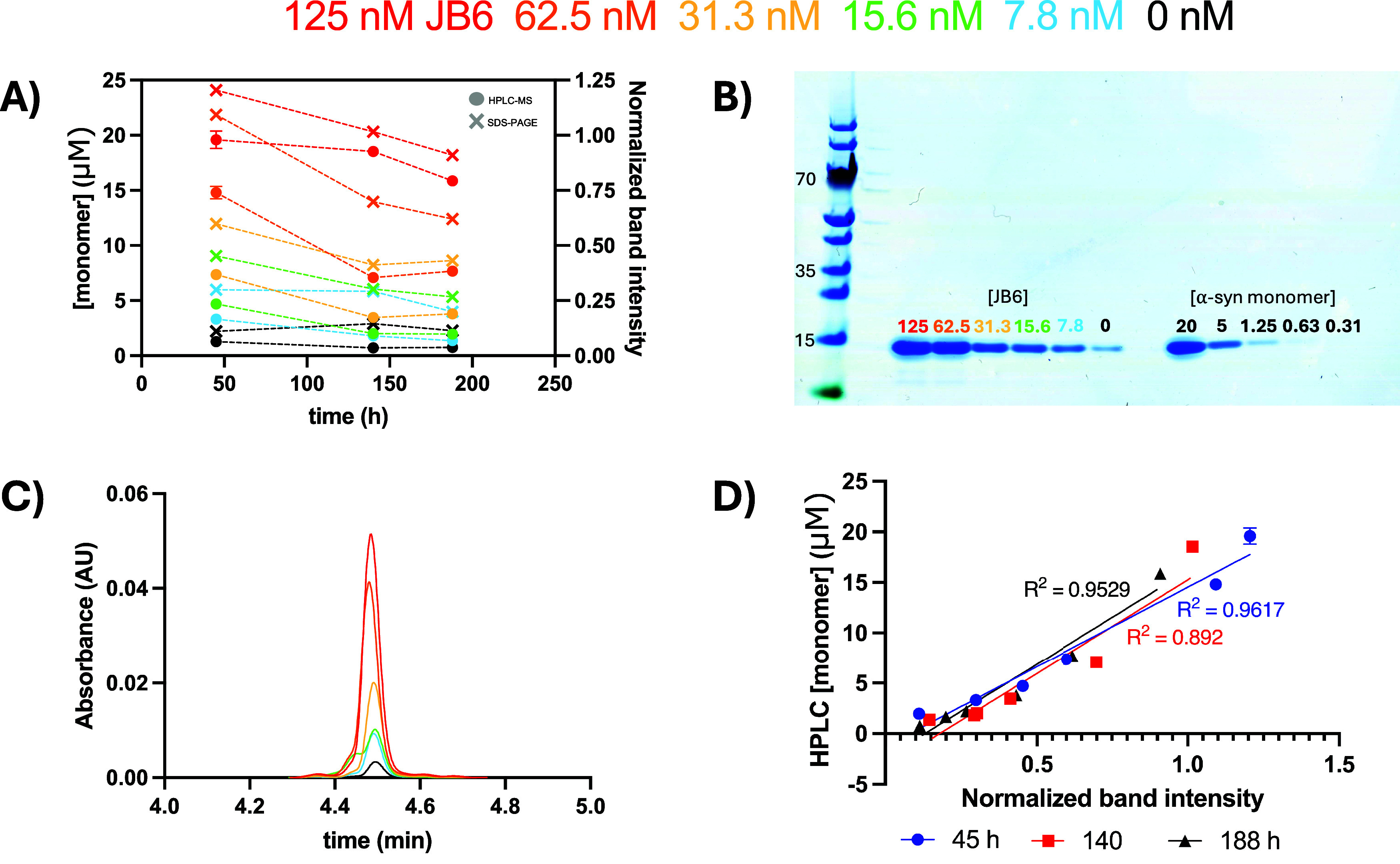
Quantification of α-syn apparent solubility at different concentrations of JB6 at pH 5.5 and 37 °C. Color coding applies to panels (A)–(C). (A) Apparent solubility of α-syn in the presence of different concentrations of JB6. The total concentration of α-syn in all samples is 20 μM. Steady state appears to be reached for all samples except for the one with 125 nM JB6 (see Figure S7 where a plateau is reached for all samples except for those with 125 nM JB6). The α-syn concentration in the supernatant was measured at three time points with two methods. Filled circles represent monomer concentration measured with absorbance using high-performance liquid chromatography-mass spectrometry (HPLC-MS). × symbols represent SDS-PAGE analysis with the intensity of α-syn in the supernatant normalized against the intensity of 20 μM α-syn monomer. (B) SDS-PAGE of supernatant samples at 45 h and standard samples of pure monomeric α-syn. Gels at 140 and 188 h are shown in Figure S9. (C) HPLC-MS trace at 280 nm of the supernatant sample at 45 h (see Figure S8 for full trace). (D) Correlation between HPLC-MS measured monomer concentration and normalized band intensity from SDS-PAGE. Data points at each time point cover all six JB6 concentrations.

### 2.8 Cryo-TEM

The samples used for the images presented in Figure 6 were prepared at pH 4.5 in a 96-well half-area low-binding PEG-coated polystyrene plate (3881, Corning) with a transparent bottom and sealed with a SealPlate film (Excel Scientific) (according to Section 2.5). Samples were collected into low-binding Eppendorf tubes at the end of the corresponding kinetic experiment by carefully pipetting up and down and scraping the bottom of the well, using a pipet tip (this was done to ensure that the aggregates that sediment and stick to the bottom of the wells were collected). The samples used for the images presented in Figure 5 were also prepared in a 96-well half-area low-binding PEG-coated polystyrene plate with a transparent bottom, sealed with a film, and incubated for 2 days. The samples were prepared at four different pH values: 4.0, 4.5, and 5.0, 10 mM acetate buffer, 0.02% NaN_3_, and pH 5.5 (10 mM MES/NaOH, 0.02% NaN_3_). The samples were collected as described above.

Specimens for cryo-TEM were prepared in an automatic plunge freezer system (Leica EM GP). The climate chamber temperature was kept at 21 °C, and relative humidity was ≥90% to minimize loss of solution during sample preparation. The specimens were prepared by placing 4 μL of solution on glow-discharged lacey Formvar carbon-coated copper grids (Ted Pella) and blotted with filter paper before being plunged into liquid ethane at −183 °C. This leads to vitrified specimens, avoiding component segmentation and rearrangement, and the formation of water crystals, thereby preserving original microstructures.

The vitrified specimens were stored under liquid nitrogen until they were imaged. A Fischione Model 2550 cryo transfer tomography holder was used to transfer the specimen into the electron microscope, JEM 2200FS, equipped with an in-column energy filter (Omega filter), which allows zero-loss imaging. The acceleration voltage was 200 kV and zero-loss images were recorded digitally with a TVIPS F416 camera using SerialEM under low dose conditions with a 10 eV energy selecting slit in place.

The diameter of the fibrils was measured at multiple different locations along different aggregates in different images. This was done by using the ruler tool in Adobe Photoshop.

### 2.9 Observation of ThT-Positive and ThT-Negative Aggregates

At the end of the kinetic experiments (10 mM MES, 1.4 mM NaP, 0.02% NaN_3_, pH 5.5, 20 μM ThT), the samples were collected and centrifuged for 30 min at 14,200*g* to separate aggregates from free monomers. The color of the pellets obtained after the centrifugation step was compared and photographed. For comparison, samples containing JB6 only at the highest concentration (2 μM) were also inspected and documented in the same way.

### 2.10 pH Dependence of DNAJB6 Aggregate Stability

The pH dependence of JB6 aggregate stability was investigated by recording the scattered light intensity at 90° while varying the pH in a Labbot instrument (Probation Laboratories Sweden AB) using laser light at 635 nm. The pH was measured continuously using an Orion star A211 pH meter connected to an Orion ROSS Combination pH Micro Electrode and the Labbot instrument. 1 mL of 2 μM JB6 in 10 mM acetate buffer at pH 4.0 was added to a Quartz SUPRASIL (HellmaAnalytics) cuvette with a path length of 10 mm. A micro stir bar (8 mm × 1.5 mm, polytetrafluoroethylene (PTFE) coated) was added to the cuvette, and the sample was continuously stirred at around 500 rpm at 25 °C. The pH value and the light scattering intensity were measured every 60 s. 0.05 μL of the titrant, 1 M NaOH, was added to the sample every 300 s. The detected light scattering is presented in arbitrary units, in which 1 unit equals 1 ppm of the detector maximum output.

### 2.11 Adsorption of JB6 to Experimental Surfaces

The absorption of 250 nM JB6 (in 10 mM MES, 1.4 mM NaP, 0.02% NaN_3_, pH 5.5) to a low-binding 96-well half-area PEG-coated polystyrene plate (3881, Corning) and NMR tubes made from borosilicate glass, Type 1 Class B standard, recognized as N51A (Wilmad-LabGlass SP ScienceWare 5 mm O.D. Thin Walled Economy NMR Tubes). 250 nM JB6 was prepared in LoBind Eppendorf Tubes. Samples were treated in an identical way and transferred to NMR tubes and plates using Henke-Ject syringe (Henke Sass Wold, Germany) attached to a 0.80 × 80 mm^2^ Sterican needle (B. Braun, Germany). 1.6 mL of sample was added into each NMR tube, and 100 μL of sample was added into each well in the plate. Samples were incubated at 37 °C for 1.5 h, 4 h, and 1 day. 100 μL of sample was taken from each replicate from the NMR tube, by not touching the walls of the NMR tube while withdrawing the sample. 20 μL was taken from the middle of each well, without touching the surfaces, and samples from three wells were combined for each replicate to reach 60 μL. Samples were analyzed by HPLC-MS, with recording of the absorbance at 205 nm (2–3 measurements per replicate) and by SDS-PAGE.

## 3 Results

### 3.1 JB6 Inhibits the Aggregation of α-syn at Mildly Acidic pH

The aggregation kinetics of 20 μM α-syn in the presence of JB6 was investigated at mildly acidic pH (pH 5.5), where secondary nucleation of α-syn is prominent.^33,35^ The concentration of JB6 ranged from 7.8 to 125 nM, corresponding to 0.04–0.6% of the α-syn molar concentration. The results show that JB6 suppresses α-syn aggregation at very low stoichiometric ratios of JB6 and α-syn, where inhibition was observed also at the lowest JB6 concentration, 7.8 nM (0.04% of the total α-syn concentration). JB6 was found to fully inhibit the amyloid fibril formation at the highest concentration (≥125 nM, i.e., ≥0.6% of the total α-syn concentration) over the experimental time frame (188 h) (see Figure 2A). The half-times of α-syn in the presence of JB6 were found to increase linearly with the JB6 concentration (see Figure 2B).

### 3.2 Change in the Apparent Solubility of α-syn as a Function of JB6 Concentration

The amount of α-syn monomer in solution after aggregation was measured with three different methods: SDS-PAGE, HPLC-MS, and NMR spectroscopy.

#### 3.2.1 The Effect on Apparent Solubility Measured by SDS-PAGE and HPLC-MS

For HPLC-MS (Figure 3A) and SDS-PAGE (Figure 3B) analysis, samples were collected at different time points from the kinetic experiment (see Figures 2 and S6), after 45, 140, and 188 h, centrifuged, and an equal volume of each supernatant was analyzed (see Section 2.6).

After 188 h (the experimental time frame), the equilibrium appears to have been reached for all samples, except for the ones supplemented with the highest JB6 concentration, 125 nM (0.6%) JB6 (see Figure S7), in which the concentration of monomer left in solution was still very high, 16 μM.

HPLC-MS allows for α-syn monomer quantification down to the nanomolar range. An exemplary HPLC trace of α-syn is shown in Figure 3C, from which the apparent solubility could be determined (the HPLC-MS traces up to 6 min, showing all detectable peaks on the chromatogram, are presented in Figure S7).

The α-syn monomer concentration left in solution, measured by HPLC-MS, was found to increase with the JB6 concentration (Figure 3A, represented with filled circles). At equilibrium, the apparent solubility was found to be higher for all samples with JB6 than for α-syn alone. The concentration of α-syn left in solution was measured as 0.8 μM for the sample containing α-syn alone, and 1.4, 2.0, 3.8, and 7.7 μM for the samples containing 7.8, 15.6, 31.3, and 62.5 nM JB6, respectively. The concentration of α-syn remaining in solution at 62.5 nM JB6 is thus a factor of 10 higher than that for α-syn alone.

The same samples as used for HPLC-MS were also analyzed by SDS-PAGE, where the signal intensities of the bands were compared and analyzed using ImageJ (a gel of samples harvested after 45 h incubation is shown in Figure 3B). SDS-PAGE of samples harvested after 140 and 188 h are shown in Figure S8. The results were consistent with the HPLC-MS analysis, showing that the concentration of α-syn in solution at steady state is higher in the presence of JB6 at all concentrations tested (7.8–125 nM or 0.04–0.6%) compared to α-syn alone, indicating an increase in α-syn solubility in the presence of JB6.

In Figure 3A, the band intensities of the gel in Figure 3B are normalized against that of 20 μM α-syn monomer (represented with × in the figure). The signal intensities above 1.0 are possibly attributed to experimental errors including minor variations in the volumes of the samples loaded onto the gel.

The monomer concentrations from the HPLC-MS correlate with the normalized band intensities from the SDS-PAGE, with *R* ^2^ above 0.89 for all three time points, 45, 140, and 188 h (see Figure 3D). This linearity remains between the first measurement at 45 h and the last one at 188 h, as evident by the *R* ^2^ values only dropping slightly from 0.96 to 0.95.

The ThT fluorescence signal intensity was affected in the presence of JB6. This can be seen in Figure 2A, where the signal intensity at the final plateau decreases with increased concentration of the JB6. The decrease in signal may in part be due to reduced fibril concentration and in part to changes in surface character of the aggregates. A lower amount of free α-syn monomer was found left in solution in the samples having a higher ThT fluorescence intensity at the final plateau. The ThT fluorescence signal intensity at the final plateau may thus at least in part report on the amount of fibrils formed.

### 3.3 The Effect of JB6 Measured Directly Using Solution-State NMR Spectroscopy

Here, we monitored the effect of JB6 on the aggregation rate of α-syn using solution-state NMR spectroscopy. This was done as a complement to the kinetic analysis presented in Section 3.1, where the formation of fibrils is indirectly monitored using the fluorescent dye ThT. The NMR signals obtained for samples consisting of monomers in the presence of the fibrils originate from the free monomer in solution, as the fibrils are NMR invisible (signals from fibrils are broadened beyond visibility). The recorded NMR spectra, thus, directly report on the concentration of free monomer in the sample during aggregation.

Samples of 20 μM ^15^N-labeled α-syn alone and in the presence of 250 nM nonlabeled JB6 were prepared in 10 mM MES, 1.4 mM NaP, 0.02% NaN_3_, pH 5.5, 1% seeds, 10 μM free ^15^N-labeled tryptophan (Trp) (Figure S9 shows no significant impact of Trp on aggregation kinetics), DSS, 10% D_2_O. As shown in Figure 4A, the measured monomer concentration was found to decrease over time in a sigmoidal manner. The retardation of the aggregation in the presence of 250 nM JB6 (1.25%) is clearly visible, with half-times of 35 and 4.9 h with and without JB6, respectively. This is consistent with the ThT kinetic data (Figure 2A) showing significant retardation of α-syn aggregation in the presence of JB6. It should be noted that in the NMR spectroscopy experiments, higher molar ratios of JB6 are needed in order to observe the same retardation effect of JB6 on α-syn aggregation as in experiments performed in low-binding plates (see Section S8 and Figure S10). This can be explained by differences in the experimental setup, such as differences in the container surface properties, in the surface-to-volume ratios, and agitation,^64^ which may affect the behavior of α-syn and JB6. We find that JB6 adsorbs to the glass surface of the NMR tubes but not to the low-binding plates used for the ThT kinetic experiments (see Section S9 and Figure S11).

**Figure 4:**
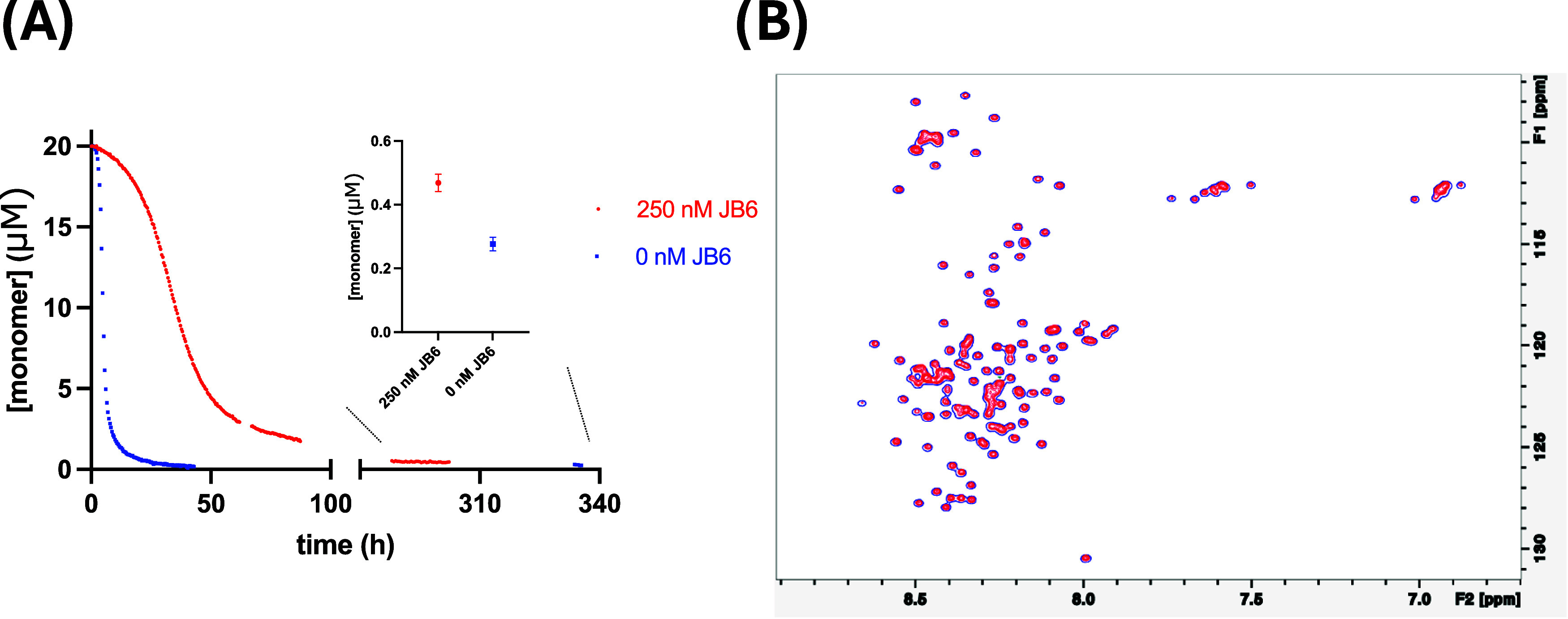
NMR data for α-syn with and without 250 nM JB6 at pH 5.5, 37 °C. (A) α-syn monomer concentration, determined through integration of the amide region in the 1D ^1^H spectra. Magnified are the mean and standard deviation (SD) of the final intensity measurements at around 300 h. (B) Superposition of the first recorded ^1^H–^15^N HSQC spectrum of each sample in panel (A) (α-syn with 250 nM JB6 in red and without JB6 in blue). F1 on the *y*-axis represents the chemical shift of ^15^N signals and F2 on the *x*-axis of the ^1^H signals. The figure was produced with TopSpin 4.3.0.

Furthermore, an increase in the apparent solubility of α-syn in the presence of JB6 was observed after about 300 h. Averaging over the integrated peak intensities shows 0.47 ± 0.027 and 0.28 ± 0.021 μM free α-syn monomers in solution with and without JB6, respectively. The detected increase in apparent solubility is consistent with the data shown in Section 3.2 (using low-binding surfaces), where we in all cases detect an increase in the apparent solubility of α-syn in the presence of JB6. The lower concentrations detected by NMR spectroscopy are most likely related to the adsorption of JB6 onto the glass surface of the NMR tubes, resulting in a lower amount of available JB6 in solution (see Figure S11).

^15^N HSQC spectra of samples consisting of 20 μM α-syn in the presence and absence of JB6 were superimposed, showing no apparent effects of 250 nM or 2 μM JB6 on the detected α-syn monomer signals (Figures 4 and S12). The superimposed ^15^N HSQC spectra at the respective *t*_1/2_ values also display no chemical shift changes in the monomer signal (Figure S12A). We would expect significant binding in the case of 10% JB6 to be visible in the NMR spectrum (Figure S12B). Furthermore, we can not explain the significant retardation with binding of JB6 to α-syn monomers at low molar ratios. This further strengthens that JB6 binds mainly to aggregated forms of α-syn, or forms coaggregates with the α-syn.

### 3.4 Evidence of Coaggregation

In this section, we present three lines of evidence for the formation of coaggregates between α-syn and JB6. First, changes in the ultrastructure were detected by cryo-TEM. Second, formation of ThT-negative aggregates was observed in the presence of JB6. Finally, we measured the depletion of JB6 from solution in the presence of α-syn, indicating the association of JB6 and α-syn.

#### 3.4.1 The Effect of JB6 on the Ultrastructure of α-syn Fibrils Detected by Cryo-TEM

The structure of α-syn fibrils in the presence of JB6 at different concentrations was investigated by cryo-TEM at pH 4.5 and 5.5. The theoretical pI value of JB6 is calculated to be 7.3 (Table 1).

**Table 1:** Theoretical pI Value of JB6^a^

	residues	theoretical pI value
whole sequence	1–241	7.3
N-terminal domain	1–71	9.4
linker	72–184	4.8
C-terminal domain	185–241	10.0

a The theoretical pI value of the whole protein sequence (UniProt: O75190-2), as well as individual parts, was calculated using the p*K*_a_ values of model compounds^65^ and the Henderson–Hasselbalch equation assuming ideal titrations. The titration of the N-terminus was taken into account for JB6 and NTD. The titration of the C-terminus was taken into account for JB6 and CTD.

The light scattering of JB6 (Figure 5) was measured at 90° as a function of pH by titrating the sample from low to high pH with 1 M NaOH (Figure 5A). Maximum scattering was observed at pH 5.5 for the JB6. Cryo-TEM images of JB6 alone were obtained at four different pH values (4.0, 4.5, 5.0, and 5.5). The cryo-TEM images did correlate well with our scattering data, showing larger structures of JB6 at pH 5.0 and 5.5 compared with those at pH 4.0 and 4.5 (Figure 5).

**Figure 5:**
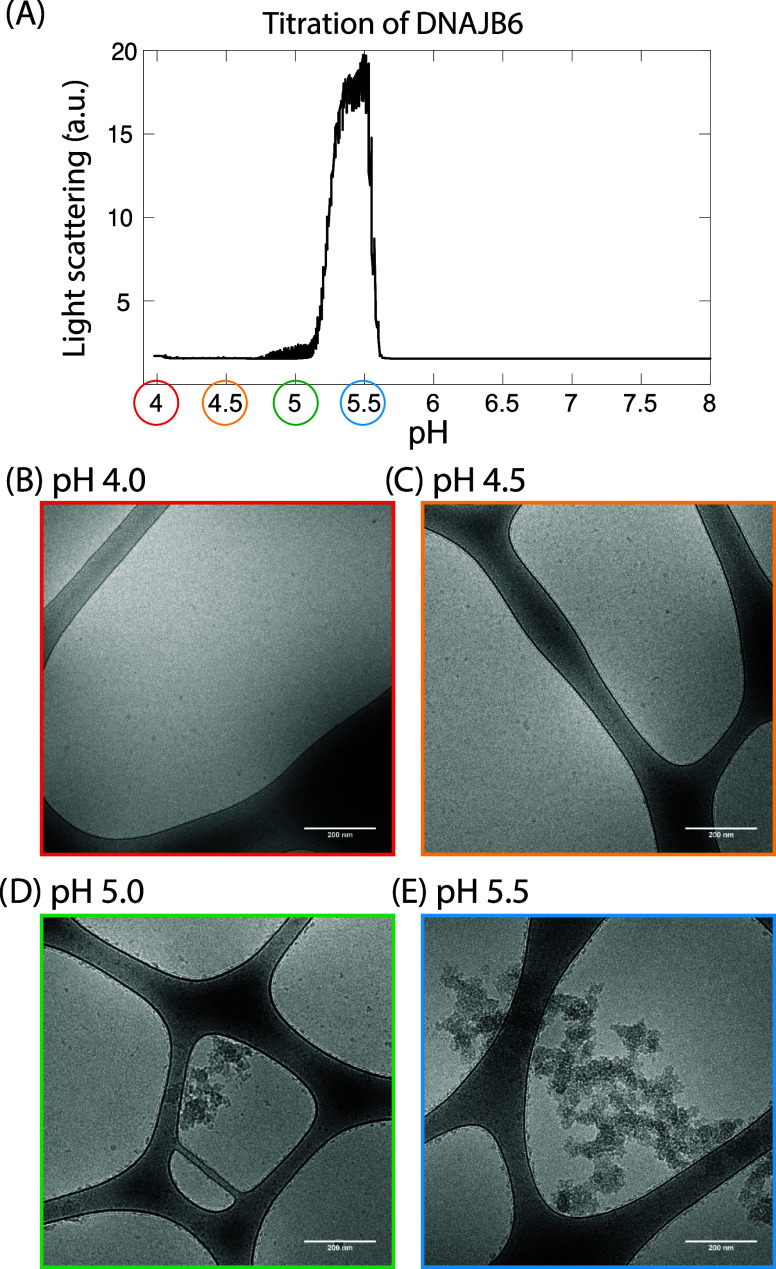
Light scattering and cryo-TEM images of JB6 alone as a function of pH. (A) Right-angle light scattering of 2 μM JB6 in 10 mM acetate buffer, 1.4 mM NaP, and 0.02% NaN_3_ titrated with 1 M NaOH. Cryo-TEM images of 2 μM JB6 at (B) pH 4.0, (C) pH 4.5, (D) pH 5.0, and (E) pH 5.5.

Furthermore, more clear cryo-TEM images of α-syn fibrils alone were obtained at pH 4.5 compared with at pH 5.5. For comparison of the ultrastructures of α-syn alone and in the presence of JB6, cryo-TEM imaging was performed at both pH 4.5 (Figures 6 and S13–S16) and pH 5.5 (see Figure S17).

**Figure 6:**
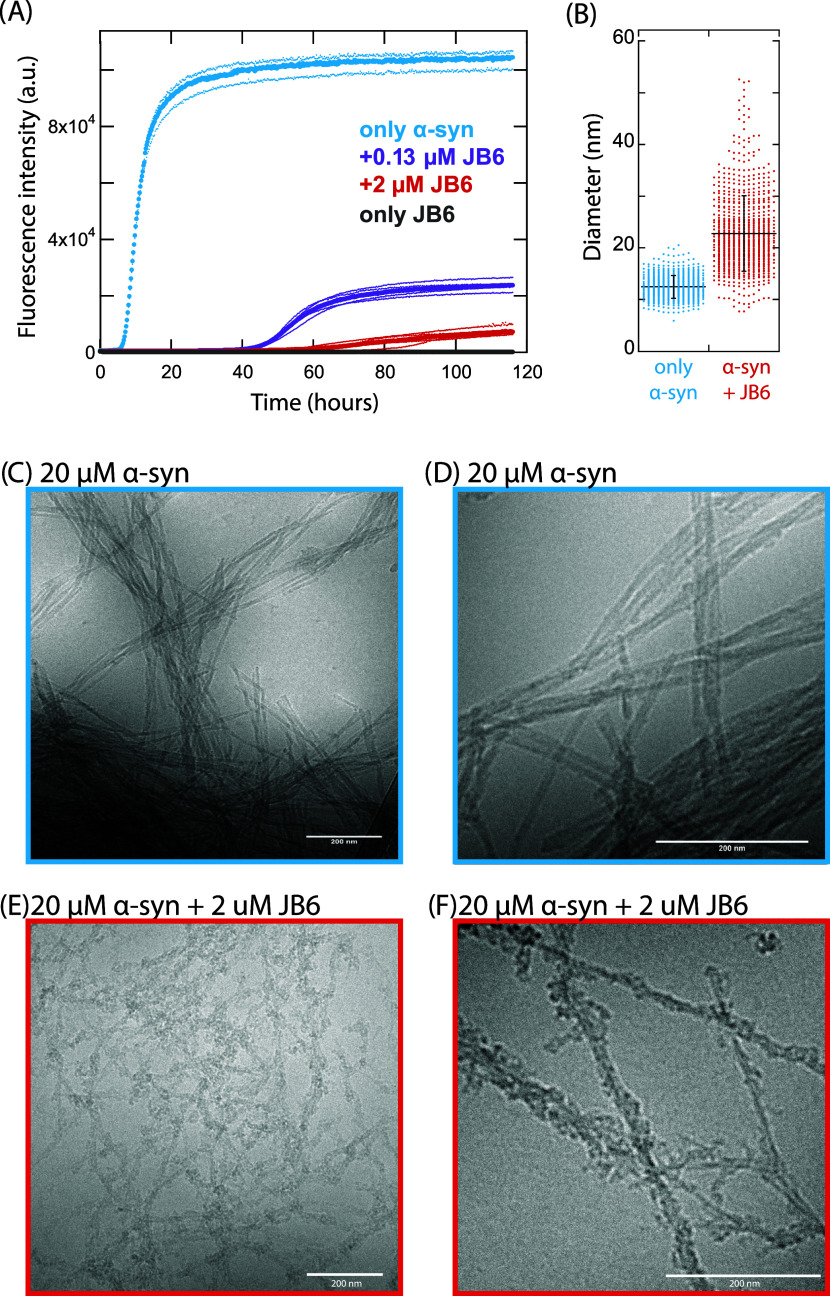
Formation of coaggregates of α-syn and JB6. Cryo-TEM and kinetics analysis of α-syn in the presence of JB6 at pH 4.5 (10 mM acetate buffer, 1.4 mM NaP, and 0.02% NaN_3_). (A) Comparison of aggregation rate and ThT fluorescence intensity between 20 μM α-syn alone (blue), and in the presence of 2 μM (10%, red) and 0.13 μM (0.6%, purple) JB6, as well as 2 μM JB6 alone (gray). (B) Average diameters of single α-syn fibrils (blue) and coaggregates of α-syn and JB6 (10%, red) are 12.5 ± 2.2 nm (*n* = 668) and 22.8 ± 7.3 nm (*n* = 765), respectively. The data is visualized with a dot plot, where each data point is represented with a dot and the average and standard deviation are shown with black lines. (C–F) Cryo-TEM images showing different ultrastructure between the samples collected at the end of the kinetic experiment: (C) 20 μM α-syn, 40k magnification. (D) 20 μM α-syn, 80k magnification. (E) 20 μM α-syn + 2 μM JB6, 40k magnification. (F) 20 μM α-syn + 20 μM JB6, 80k magnification. The scale bars correspond to 200 nm.

JB6 suppresses the aggregation of α-syn at pH 4.5 (Figure 6), leading to an overall lower aggregation rate, consistent with the results presented above for pH 5.5 (Figures 2 and S17). Samples were collected at the end of the experiment and analyzed with cryo-TEM. The sample containing α-syn alone shows a large amount of bundled-up and straight fibrils (see Figures 6C,D, S13, and S18), consistent with previous observations for α-syn at mildly acidic pH.^66^

The samples containing 2 μM JB6 alone at pH 4.5 show small objects with a diameter of 6.2 ± 1.1 nm (*n* = 60), potentially indicative of JB6 oligomers (see Figures 5 and S14).

The images of α-syn in the presence of 2 μM (10%) JB6 show aggregates that have remarkably different ultrastructures compared to α-syn alone (see Figures 6E,F and S15). The aggregates formed in the presence of JB6 are elongated fibrillar aggregates; they appear less bundled and have varying thickness. The fibrils are more irregular, with small knobs along the fibril structures. These results strongly indicate that coaggregates of α-syn and JB6 are formed. The average diameter of single fibrils of α-syn is smaller than for the coaggregates of α-syn and JB6, with a diameter of 12.5 ± 2.2 nm (*n* = 668) and 22.8 ± 7.3 nm (*n* = 765), respectively (see Figure 6B). In the presence of JB6 at the lower concentration, 125 nM (0.6%) (Figure S16), both types of structures are detected, corresponding to the images containing α-syn alone (Figure 6C,D) and in the presence of 2 μM (10%) JB6 (coaggregates, Figure 6E,F).

#### 3.4.2 Detection of ThT-Negative Aggregates

The samples containing both α-syn and JB6 at high stoichiometric ratios of chaperone to client (showing no increase in ThT fluorescence) formed aggregates that were ThT-negative. This was investigated by collecting the samples from the plate wells into Eppendorf tubes at the end of a kinetic experiment, followed by centrifugation. After 2 days of incubation at 37 °C, the samples containing 20 μM α-syn alone or JB6 at low stoichiometric ratios to α-syn (20 μM α-syn and 16 nM JB6) showed an increase in the ThT fluorescence intensity and had reached the final plateau (see Figure 7E). After centrifugation, the corresponding samples consisted of supernatant and pellets of a bright yellow color, indicative of ThT binding (see Figure 7A,B). On the other hand, the samples that contained higher stoichiometric ratios of JB6 to α-syn (20 μM α-syn and 2 μM JB6) showed no increase in ThT fluorescence after 2 days of incubation at 37 °C. Despite that, after centrifugation, the samples were separated into supernatant and a pellet. The corresponding pellets were white and also smaller than the yellow pellets in the other samples (Figure 7C). This indicates that JB6 associates with α-syn, forming coaggregates that do not enhance ThT fluorescence to the same extent as that of the pure α-syn fibrils. This is consistent with the cryo-TEM images showing that aggregates are present in the samples of the higher stoichiometric ratios of JB6 to α-syn that have no or a low increase in the ThT fluorescence intensity (Figures 6E,F, S15, and S17B (3 and 10% JB6)). For comparison, samples containing JB6 alone were treated in the same manner and showed no visible pellets (see Figure 7D), further supporting the conclusion of the formation of ThT-negative coaggregates of α-syn and DNAJB6.

**Figure 7:**
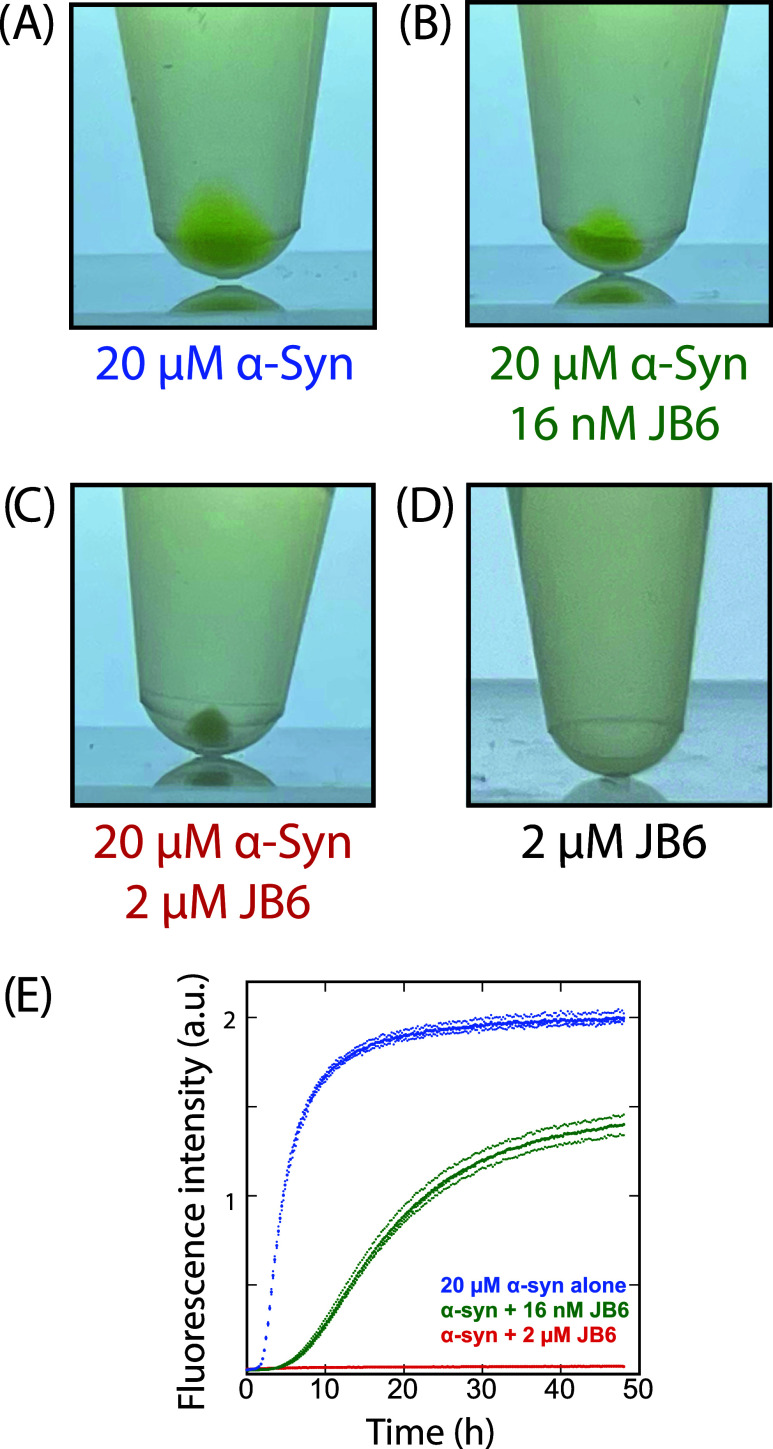
ThT-negative coaggregates. Samples consisted of 20 μM α-syn in the absence and presence of JB6 (2 μM and 16 nM), and 2 μM JB6 alone in 10 mM MES, 1.4 mM NaPB, 0.02% NaN_3_ at pH 5.5 with 20 μM ThT. Samples were collected after 2 days of incubation (48 h) at 37 °C in low-binding plates without shaking, centrifuged, and inspected, where the appearance of the pellets was compared. (A) 20 μM α-syn, showing a bright yellow pellet. (B) 20 μM α-syn with 16 nM JB6, showing a bright yellow pellet. (C) 20 μM α-syn with 2 μM JB6, showing a white pellet, (D) 2 μM JB6 alone, showing no pellet. (E) Kinetic traces of the corresponding samples. The average of 3–4 replicates is shown in bold.

#### 3.4.3 Depletion of JB6 from Solution

In parallel to the samples of α-syn and JB6 at pH 4.5 being analyzed by cryo-TEM imaging (see Figure 6), the same samples were centrifuged and the supernatant was measured by HPLC-MS to compare the amount of α-syn and JB6 in the supernatant. We detected depletion of JB6 from the solution in the presence of α-syn based on the HPLC trace (Figure 8A). For comparison, samples of JB6 alone at pH 4.5 were centrifuged, and the supernatant was analyzed by SDS-PAGE and compared to a sample that had not been centrifuged (see Section S13). The result shows that JB6 in the absence of α-syn stays in solution at pH 4.5 (see Figure S18). This is supported by HPLC-MS analysis, showing that JB6 stays in solution in the absence of α-syn (Figure 8A). This, in conjunction with the results presented in Sections 3.4.1 and 3.4.2, shows that larger sedimenting structures, possibly coaggregates of JB6 and α-syn, are formed in the samples consisting of α-syn and JB6, while no such structures are formed in the samples consisting of JB6 alone, where JB6 stays in solution.

**Figure 8:**
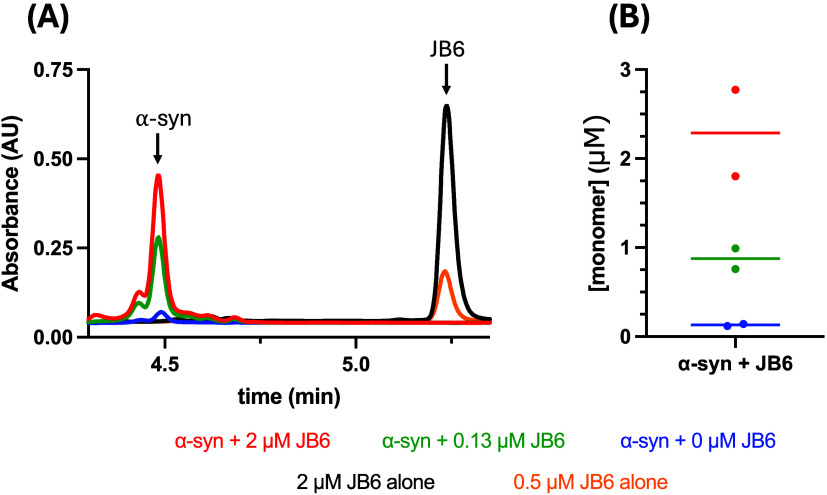
HPLC-MS analysis of α-syn samples aggregated without or with JB6 at different concentrations. The conditions are the same as those in Figure 6A at pH 4.5. (A) HPLC-MS traces monitored with the absorbance at 205 nm after injecting supernatants, with the color code below showing the initial total sample compositions. The peaks at 4.5 and 5.2 min correspond to α-syn and JB6, respectively. (B) α-syn concentration in the supernatant as derived from the integration of the peak at 4.5 min.

Furthermore, we detected an increase in the apparent solubility of α-syn in the presence of JB6 at pH 4.5 (Figure 8B), consistent with the results at pH 5.5 (Figures 3 and 4). The mean apparent solubility was 2.3, 0.88, and 0.13 μM at 2, 0.12, and 0 μM JB6, respectively.

## 4 Discussion

Extensive research has been devoted to identifying and characterizing substances that inhibit the formation of amyloid fibrils. These inhibitors encompass a variety of compounds, including small molecules, antibodies, and molecular chaperones.^55,67–69^ In this study, we obtain strong indications that the molecular chaperone JB6 affects the fibril formation of α-syn, in terms of both kinetics, by retarding aggregation, and thermodynamics, by increasing the apparent solubility of α-syn. This will be further discussed in the following sections.

### 4.1 Suppression of Amyloid Formation

In this work, we show that the amyloid formation of α-syn is suppressed by JB6 in vitro in a concentration-dependent manner at low stoichiometric ratios at pH 5.5, with molar ratios of JB6 to α-syn down to 0.0004:1 (Figure 2).

In the kinetic experiments presented in Figure 2, the aggregation was fully suppressed at the higher stoichiometric ratios within the time frame tested. Similar results have previously been obtained for polyglutamine peptide^13^ and amyloid β peptide.^14,15,48^ Chaperones have been found to affect the different microscopic steps of amyloid fibril formation in a system-specific manner, depending on the identity of the chaperone and the amyloid-forming protein. JB6 has previously been found to both inhibit primary and secondary nucleation events of the amyloid fibril formation of amyloid β peptide and polyglutamine peptide, with a stronger effect on the primary nucleation.^13–16,48,55^ The effects of JB6 on nucleation are reported to be due to interactions with oligomers (or the formation of chaperon-client co-oligomers), which may stabilize the oligomers and prevent their structural conversion and nucleation to amyloid structures.^14,48,55^ Substoichiometric concentrations of the Brichos domain from proSP-C have been found to strongly interfere with the secondary nucleation of amyloid β peptide.^57^ The cochaperonin prefoldin has also been found to interfere with the secondary nucleation as well as the elongation of IAPP.^70^ A study using a mixture of crystallins showed suppression of aggregation of α-syn aggregation at pH 5.5, indicating interaction between crystallins and α-syn fibrils, further suggesting inhibition of elongation and secondary nucleation steps.^71^

The results presented here were performed under conditions (mildly acidic pH, quiescent, seeded, nonbinding PEGylated plates) where effects on secondary nucleation are visible and primary nucleation is a rare event.^33^ The significant and efficient suppression under these conditions, therefore, indicates that JB6 may interfere with the secondary nucleation events of α-syn amyloid fibril formation.

### 4.2 Evidence of the Formation of Coaggregates between α-syn and JB6

In this study, we have three lines of evidence for the formation of coaggregates between α-syn and JB6.

Firstly, cryo-TEM analysis revealed a striking difference in the mesoscopic structures of the α-syn aggregates formed in the presence and absence of JB6 (Figures 6 and S13–S17). To our knowledge, this is the first structural indication of a coaggregate of an amyloid-forming protein and a chaperone.

Secondly, ThT-negative aggregates are detected in the samples consisting of α-syn and JB6 at higher molar ratios (≥3% JB6). This was detected by visually inspecting centrifuged samples (white pellets, Figure 7), although aggregates were clearly seen by cryo-TEM. This suggests that the coaggregates of α-syn and JB6 are structurally different from the pure α-syn aggregates.

Thirdly, we show that JB6 is depleted from solution upon incubation in the presence of α-syn (Figure 8), indicating interaction between JB6 and α-syn and formation of larger structures that sediment upon centrifugation.

The results presented here are in good agreement with previous studies of Aβ42 and JB6 that showed association between JB6 and the fibrils with immunoblots.^14,15^

### 4.3 Formation of Coaggregates and Increase in Apparent Solubility

An increase in apparent solubility of an amyloid protein in the presence of JB6 has been reported for Aβ42 ^15^ and is here observed for α-syn. The concentration of the free α-syn monomer in solution in the end state was found to positively correlate with the chaperone concentration. This was seen by comparing the amount of monomer in the supernatant over sedimented aggregates using different methods: SDS-PAGE, NMR spectroscopy, and HPLC-MS with absorbance at 280 and 205 nm. Our experimental data thus provide a second clear case, after Aβ42,^15^ for which the presence of JB6 leads to a significant increase in amyloid peptide apparent solubility.

A change in the solubility, an equilibrium parameter, requires a thermodynamic explanation. One possible explanation was recently put forward^9^ in which the chaperone has a high chemical potential on its own, which it can reduce upon formation of coaggregates with an amyloid peptide. The reduction in chaperone chemical potential could thereby compensate for an increase in the chemical potential of the amyloid peptide relative to pure aggregates. This would allow the system as a whole to lower its free energy in spite of an increase in the contribution from the amyloid peptide and provide a driving force for an increased amyloid solubility in the presence of the chaperone.^9^

As a consequence of the second law of thermodynamics, a substance will have equal chemical potential in all phases in a system at thermodynamic equilibrium. Thus, higher amyloid peptide concentration (higher chemical potential) in the solution phase could be supported by a higher chemical potential of the amyloid peptide in the aggregate phase, implying that these must be altered in some way (Figure 9).^9^

**Figure 9:**
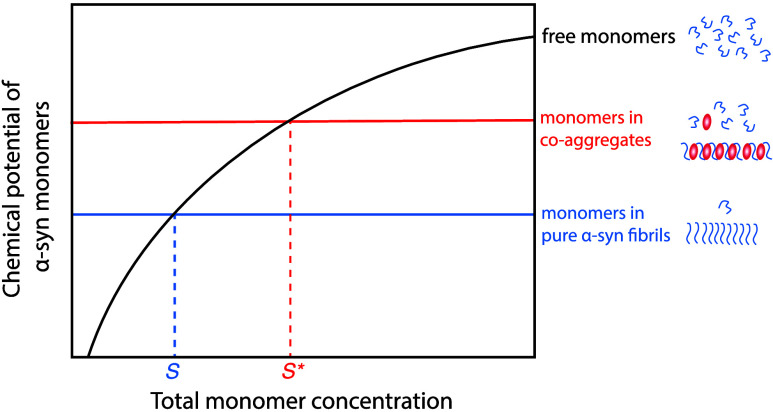
Schematic illustration of the chemical potential of α-synuclein monomers free in solution (black line), within coaggregates (red line), and within pure α-syn fibrils. The chemical potential is shown as a function of the total monomer concentration. The solubility of α-syn within pure α-syn fibrils (*S*) and within coaggregates (S*) is highlighted with dashed lines.

The chemical potential of free monomers in solution (μ_m_) can be approximated by its intrinsic energy ε_m_ and the term coming from the entropy of mixing:

$$\;\mu_{m}=\epsilon_{m}+\mathrm{RT}\;\ln\left[m\right]$$

where [m] is the concentration of monomers in solution.

Amyloid fibrils are large and highly ordered structures and can therefore be seen as a solid phase. The contribution of translation entropy is minimal, and the chemical potential of monomers in fibrils (mf) can be approximated to equal the intrinsic energy:

$$\mu_{mf}=\epsilon_{\mathrm{mf}}$$

The chemical potential of the free monomers in solution is thus concentration-dependent, and for monomers in fibrils, concentration-independent (Figure 9). At equilibrium, the chemical potential of the monomers in solution is equal to that of the monomers in the fibrils. The solubility, *S*, is equal to the concentration of monomers in solution at equilibrium and can thus be written as

$$S=\exp\left(\frac{\epsilon_{\mathrm{mf}}-\epsilon_{m}}{\mathrm{RT}}\right)$$

The solubility of the amyloid monomers is therefore dependent on the stability of the fibrils formed, consistent with a previous study of two chemically identical α-syn fibrils of different morphology, with α-syn displaying a lower solubility in the presence of the more stable fibrils.^72^

The NMR spectra do not report on any significant binding between α-syn monomers and JB6 (see Section 3.4) in agreement with previous reports.^14,48^ Similarly, no signs of binding between α-syn monomers and the 14-3-3-*t* chaperone have been detected.^73^ Therefore, in a dilute system, we assume that the intrinsic energy of the monomer is unaltered in the presence of JB6. The chemical potential of α-syn monomer in solution in the presence of the chaperone (JB6) can thus be written as

$$\mu_{m*}=\epsilon_{m}+\mathrm{RT}\;\ln\left[m^{*}\right]$$

where m* denotes the amyloid protein monomer in the presence of a chaperone. At equilibrium, the chemical potential of monomers free in solution must be equal to the chemical potential of the monomers within the coaggregates:

$$\mu_{m*}=\mu_{\mathrm{mf}*}$$

The solubility in the presence of JB6, S*, can thus be written as

$$S^{*}=\exp\left(\frac{\epsilon_{\mathrm{mf}*}-\epsilon_{m*}}{\mathrm{RT}}\right)$$

The results presented in this paper indicate that the apparent solubility of α-syn increases in the presence of the chaperone:

$$S^{*}>S$$

which suggests that the intrinsic energy of the monomer in the fibrils formed in the presence of chaperone is higher than the intrinsic energy of the free monomer in fibrils formed from α-syn alone.

$$\epsilon_{mf*}>\epsilon_{mf}$$

This further implies that the aggregates formed in the presence and absence of the chaperone are of different structures with different stability. This is supported by our cryo-TEM results showing remarkably different structures for the fibrils formed in the absence and presence of JB6, indicating the formation of coaggregates in the latter case.

The increased apparent solubility of α-syn in the presence of JB6 thus suggests that the coaggregates are less stable structures compared to the pure α-syn fibrils.

### 4.4 The Driving Force for Coaggregate Formation between α-syn and JB6

What would be the driving force for the system to form the coaggregates in which the α-syn chemical potential is increased compared to pure α-syn aggregates? This question might be explained by the high chemical potential of the chaperone alone (Figure 10) and the requirement for the system as a whole to lower its free energy for a process to happen. At equilibrium, the system has reached the lowest possible total free energy. In the case of the chaperone, the formation of coaggregates results in a decrease in free energy that is greater than the increase in free energy of α-syn, causing the process of forming coaggregates of α-syn and JB6 to be favorable (Figure 10). Thus, the free energy of a system consisting of coaggregates is lower than for a system consisting of pure α-syn coexisting with JB6 (Figure 10).^9^ A comparable phenomenon is observed and utilized within pharmaceutical science, where an increase in solubility of a drug molecule is obtained by cocrystallization with another compound of high chemical potential, forming a structurally different cocrystal that is less stable than the pure drug crystal. The cocrystallization results in the system having overall lower free energy, where the energy loss of the drug molecule is compensated by the energy gain of the other compound.^74,75^ If on the contrary, the chaperone would bind to fibrils after their formation, as in the case of Brichos and prefoldin,^57,70^ such coating would rather stabilize the fibrils, and could only maintain or reduce the solubility.

**Figure 10:**
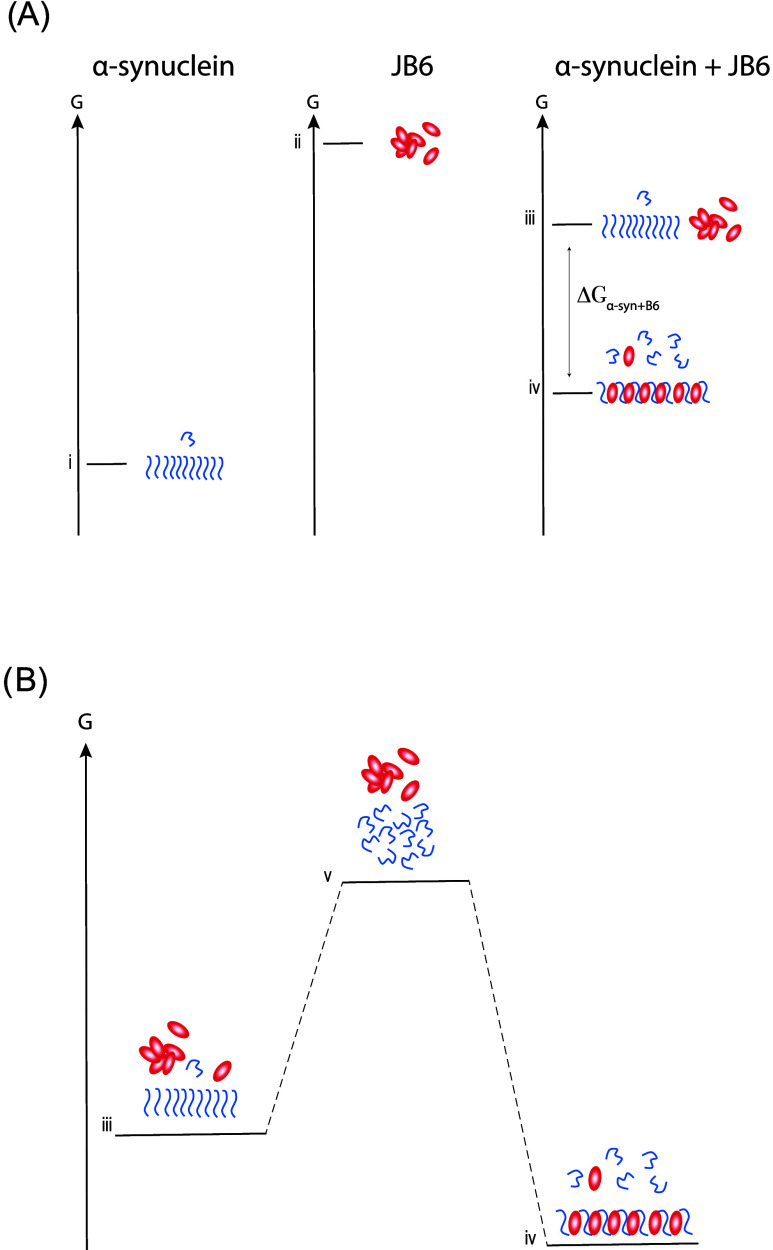
Thermodynamics behind the formation of coaggregates of α-syn and JB6. (A) Free energy diagrams of closed systems of α-syn alone (i), JB6 alone (ii), and α-syn + JB6 (iii, iv). Due to the high free energy of the chaperone alone (ii), the formation of coaggregates (iv) of α-syn and JB6 is thermodynamically more favorable than the coexistence of pure α-syn fibrils and JB6 (iii). The formation of coaggregates (iv) results in the decrease in free energy of JB6 being greater than the increase in free energy of α-syn, causing the total free energy of the system to be lower. The higher free energy of α-syn within a coaggregate (iv) than within a pure α-syn fibril (i) can explain the increase in solubility of α-syn in the presence of chaperone (iv). (B) Free energy diagram showing that formation of coaggregates (iv) from a solution consisting of α-syn and JB6 (v) is the most energetically favorable state, despite the solubility of α-syn increasing.

## 5 Conclusions

The results of this study show that the fibril formation of α-syn is suppressed by JB6 at low stoichiometric ratios (0.0004:1 molar ratio of JB6 to α-syn) in vitro at mildly acidic pH and low seed concentration, indicating that JB6 may interfere with the secondary nucleation events of α-syn amyloid fibril formation. In the presence of JB6, α-syn forms coaggregates of altered structure and stability, resulting in an increase in the apparent solubility compared to pure α-syn fibrils. A solubility increase in the presence of a chaperone can be explained by the high chemical potential of the chaperone alone, making it thermodynamically more favorable to form a coaggregate between α-syn and JB6. This free energy gain is greater than the free energy loss for α-syn, lowering the free energy of the system as a whole.

## Supplementary Material



## Notes

DNA and protein sequence of wild-type α-syn; verification of the expression of α-syn; purification of α-syn; DNA and protein sequence of DNAJB6b (JB6); purity of DNAJB6b batches used in this study; size exclusion and kinetic analysis; solubility measurements of α-syn with JB6 at pH 5.5; NMR experiments at low α-syn/JB6 molar ratio; adsorption of JB6 to NMR tubes; effect of JB6 on residue resolved signals of free α-syn monomer measured by NMR spectroscopy; cryo-TEM of α-syn in the presence of JB6 at pH 4.5; cryo-TEM and corresponding kinetics at pH 5.5; and JB6 in supernatant at pH 4.5 (PDF)

## References

[1] Sipe J. D., Benson M. D., Buxbaum J. N., Ikeda S. I., Merlini G., Saraiva M. J., Westermark P. (2012). Amyloid fibril protein nomenclature:
2012 recommendations from the Nomenclature Committee of the International
Society of Amyloidosis. Amyloid.

[2] Lansbury P. T., Lashuel H. A. (2006). A century-old debate
on protein aggregation and neurodegeneration
enters the clinic. Nature.

[3] Chiti F., Dobson C. M. (2006). Protein misfolding,
functional amyloid, and human disease. Annu.
Rev. Biochem..

[4] Bukau B., Weissman J., Horwich A. (2006). Molecular Chaperones and Protein
Quality Control. Cell.

[5] Lee S.-Y., Tsai F. T. (2005). Molecular chaperones
in protein quality control. BMB Rep..

[6] Cortez L., Sim V. (2014). The therapeutic potential
of chemical chaperones in protein folding
diseases. Prion.

[7] Chaplot K., Jarvela T. S., Lindberg I. (2020). Secreted Chaperones
in Neurodegeneration. Front. Aging Neurosci..

[8] Sinnige T., Yu A., Morimoto R. I. (2020). Challenging Proteostasis: Role of the Chaperone Network
to Control Aggregation-Prone Proteins in Human Disease. Adv. Exp. Med. Biol..

[9] Linse S., Thalberg K., Knowles T. P. (2021). The unhappy chaperone. QRB Discovery.

[10] Hageman J., Rujano M. A., van Waarde M. A., Kakkar V., Dirks R. P., Govorukhina N., Oosterveld-Hut H. M., Lubsen N. H., Kampinga H. H. (2010). A DNAJB
Chaperone Subfamily with HDAC-Dependent Activities Suppresses Toxic
Protein Aggregation. Mol. Cell.

[11] Nillegoda N. B., Kirstein J., Szlachcic A., Berynskyy M., Stank A., Stengel F., Arnsburg K., Gao X., Scior A., Aebersold R., Guilbride D. L., Wade R. C., Morimoto R. I., Mayer M. P., Bukau B. (2015). Crucial HSP70
co-chaperone complex unlocks metazoan protein disaggregation. Nature.

[12] Gao X., Carroni M., Nussbaum-Krammer C., Mogk A., Nillegoda N. B., Szlachcic A., Guilbride D. L., Saibil H. R., Mayer M. P., Bukau B. (2015). Human Hsp70 Disaggregase Reverses Parkinson’s-Linked *α*-Synuclein Amyloid Fibrils. Mol. Cell.

[13] Månsson C., Kakkar V., Monsellier E., Sourigues Y., Härmark J., Kampinga H. H., Melki R., Emanuelsson C. (2014). DNAJB6 is
a peptide-binding chaperone which can suppress amyloid fibrillation
of polyglutamine peptides at substoichiometric molar ratios. Cell Stress Chaperones.

[14] Månsson C., Arosio P., Hussein R., Kampinga H. H., Hashem R. M., Boelens W. C., Dobson C. M., Knowles T. P., Linse S., Emanuelsson C. (2014). Interaction of the molecular chaperone
DNAJB6 with
growing amyloid-beta 42 (A*β*42) aggregates leads
to sub-stoichiometric inhibition of amyloid formation. J. Biol. Chem..

[15] Månsson C., Van Cruchten R. T., Weininger U., Yang X., Cukalevski R., Arosio P., Dobson C. M., Knowles T., Akke M., Linse S., Emanuelsson C. (2018). Conserved S/T Residues of the Human
Chaperone DNAJB6 Are Required for Effective Inhibition of A*β*42 Amyloid Fibril Formation. Biochemistry.

[16] Gillis J., Schipper-Krom S., Juenemann K., Gruber A., Coolen S., Van Den Nieuwendijk R., Van Veen H., Overkleeft H., Goedhart J., Kampinga H. H., Reits E. A. (2013). The DNAJB6 and DNAJB8
protein chaperones prevent intracellular aggregation of polyglutamine
peptides. J. Biol. Chem..

[17] Österlund N., Frankel R., Carlsson A., Thacker D., Karlsson M., Matus V., Gräslund A., Emanuelsson C., Linse S. (2023). The C-terminal domain of the antiamyloid
chaperone DNAJB6 binds to
amyloid-*β* peptide fibrils and inhibits secondary
nucleation. J. Biol. Chem..

[18] Liberek K., Lewandowska A., Zitkiewicz S. (2008). Chaperones in control of protein
disaggregation. EMBO J..

[19] George J. M. (2001). The synucleins. Genome Biol..

[20] Burré J. (2015). The synaptic
function of *α*-synuclein. J. Parkinson’s Dis..

[21] Ulmer T. S., Bax A., Cole N. B., Nussbaum R. L. (2005). Structure and Dynamics of Micelle-bound
Human *α*-Synuclein. J.
Biol. Chem..

[22] Cho M. K., Nodet G., Kim H. Y., Jensen M. R., Bernado P., Fernandez C. O., Becker S., Blackledge M., Zweckstetter M. (2009). Structural
characterization of *α*-synuclein in an aggregation
prone state. Protein
Sci..

[23] Croke R. L., Patil S. M., Quevreaux J., Kendall Da., Alexandrescu aT. (2011). NMR determination
of pKa values in *α*-synuclein. Protein Sci..

[24] Makasewicz K., Linse S., Sparr E. (2024). Interplay of *α*-synuclein with Lipid Membranes: Cooperative Adsorption, Membrane
Remodeling and Coaggregation. JACS Au.

[25] Cheng F., Vivacqua G., Yu S. (2011). The role of
alpha-synuclein in neurotransmission
and synaptic plasticity. J. Chem. Neuroanat..

[26] Serratos I. N., Hernández-Pérez E., Campos C., Aschner M., Santamaría A. (2022). An Update
on the Critical Role of *α*-Synuclein in Parkinson’s
Disease and Other Synucleinopathies:
from Tissue to Cellular and Molecular Levels. Mol. Neurobiol..

[27] Bernal-Conde L. D., Ramos-Acevedo R., Reyes-Hernández M. A., Balbuena-Olvera A. J., Morales-Moreno I. D., Argüero-Sánchez R., Schüle B., Guerra-Crespo M. (2020). Alpha-Synuclein Physiology and Pathology:
A Perspective on Cellular Structures and Organelles. Front. Neurosci..

[28] Goedert M. (2001). Alpha-synuclein
and neurodegenerative diseases. Nat. Rev. Neurosci..

[29] Rabe M., Soragni A., Reynolds N. P., Verdes D., Liverani E., Riek R., Seeger S. (2013). On-surface
aggregation of *α*-synuclein at nanomolar concentrations
results in
two distinct growth mechanisms. ACS Chem. Neurosci..

[30] Campioni S., Carret G., Jordens S., Nicoud L., Mezzenga R., Riek R. (2014). The presence of an
air-water interface affects formation and elongation
of *α*-synuclein fibrils. J. Am. Chem. Soc..

[31] Vácha R., Linse S., Lund M. (2014). Surface effects on aggregation kinetics
of amyloidogenic peptides. J. Am. Chem. Soc..

[32] Pronchik J., He X., Giurleo J. T., Talaga D. S. (2010). In vitro formation of amyloid from *α*-synuclein is dominated by reactions at hydrophobic
interfaces. J. Am. Chem. Soc..

[33] Buell A. K., Galvagnion C., Gaspar R., Sparr E., Vendruscolo M., Knowles T. P. J., Linse S., Dobson C. M. (2014). Solution conditions
determine the relative importance of nucleation and growth processes
in *α*-synuclein aggregation. Proc. Natl. Acad. Sci. U.S.A..

[34] Uversky V. N., Li J., Fink A. L. (2001). Evidence
for a Partially Folded Intermediate in *α*-Synuclein
Fibril Formation. J. Biol. Chem..

[35] Gaspar R., Meisl G., Buell A. K., Young L., Kaminski C. F., Knowles T. P., Sparr E., Linse S. (2017). Acceleration of *α*-synuclein aggregation. Amyloid.

[36] Wu M. M., Grabe M., Adams S., Tsien R. Y., Moore H. P. H., Machen T. E. (2001). Mechanisms of pH Regulation in the
Regulated Secretory
Pathway. J. Biol. Chem..

[37] Casey J. R., Grinstein S., Orlowski J. (2010). Sensors and regulators of intracellular
pH. Nat. Rev. Mol. Cell Biol..

[38] Colomer V., Kicska G. A., Rindler M. J. (1996). Secretory
granule content proteins
and the luminal domains of granule membrane proteins aggregate in
vitro at mildly acidic pH. J. Biol. Chem..

[39] Deshayes N., Arkan S., Hansen C. (2019). The molecular
chaperone DNAJB6, but
Not DNAJB1, suppresses the seeded aggregation of alpha-synuclein in
cells. Int. J. Mol. Sci..

[40] Zarouchlioti C., Parfitt D. A., Li W., Gittings L. M., Cheetham M. E. (2018). DNAJ Proteins
in neurodegeneration: Essential and protective factors. Philos. Trans. R. Soc., B.

[41] Qiu X. B., Shao Y. M., Miao S., Wang L. (2006). The diversity of the
DnaJ/Hsp40 family, the crucial partners for Hsp70 chaperones. Cell. Mol. Life Sci..

[42] Durrenberger P. F., Filiou M. D., Moran L. B., Michael G. J., Novoselov S., Cheetham M. E., Clark P., Pearce R. K., Graeber M. B. (2009). DnaJB6
is present in the core of Lewy bodies and is highly up-regulated in
Parkinsonian astrocytes. J. Neurosci. Res..

[43] Hentze J., Folke J., Aznar S., Nyeng P., Brudek T., Hansen C. (2024). DNAJB6 is expressed
in neurons and oligodendrocytes
of the human brain. Glia.

[44] Arkan S., Ljungberg M., Kirik D., Hansen C. (2021). DNAJB6 suppresses alpha-synuclein
induced pathology in an animal model of Parkinson’s disease. Neurobiol. Dis.

[45] Aprile F. A., Källstig E., Limorenko G., Vendruscolo M., Ron D., Hansen C. (2017). The molecular chaperones
DNAJB6 and Hsp70 cooperate
to suppress *α*-synuclein aggregation. Sci. Rep..

[46] Folke J., Arkan S., Martinsson I., Aznar S., Gouras G., Brudek T., Hansen C. (2021). DNAJB6b is
Downregulated in Synucleinopathies. J. Parkinson’s
Dis..

[47] Aslam M., Kandasamy N., Ullah A. (2021). Putative second hit
rare genetic variants in families with seemingly GBA-associated Parkinson’s
disease. npj Genomic Med..

[48] Österlund N., Lundqvist M., Ilag L. L., Gräslund A., Emanuelsson C. (2020). Amyloid-*β* oligomers are captured
by the DNAJB6 chaperone: Direct detection of interactions that can
prevent primary nucleation. J. Biol. Chem..

[49] Udan-Johns M., Bengoechea R., Bell S., Shao J., Diamond M. I., True H. L., Weihl C. C., Baloh R. H. (2014). Prion-like nuclear
aggregation of TDP-43 during heat shock is regulated by HSP40/70 chaperones. Hum. Mol. Genet..

[50] Carlsson A., Olsson U., Linse S. (2023). On the micelle formation of DNAJB6b. QRB Discovery.

[51] Karamanos T. K., Tugarinov V., Clore G. M. (2019). Unraveling the structure and dynamics
of the human DNAJB6b chaperone by NMR reveals insights into Hsp40-mediated
proteostasis. Proc. Natl. Acad. Sci. U.S.A..

[52] Jumper J., Evans R., Pritzel A. (2021). Highly accurate protein
structure prediction with AlphaFold. Nature.

[53] Kakkar V., Månsson C., de Mattos E. (2016). The S/T-Rich Motif in
the DNAJB6 Chaperone Delays Polyglutamine Aggregation and the Onset
of Disease in a Mouse Model. Mol. Cell.

[54] Carlsson A., Axell E., Emanuelsson C., Olsson U., Linse S. (2024). The Ability
of DNAJB6b to Suppress Amyloid Formation Depends on the Chaperone
Aggregation State. ACS Chem. Neurosci..

[55] Arosio P., Michaels T. C., Linse S., Månsson C., Emanuelsson C., Presto J., Johansson J., Vendruscolo M., Dobson C. M., Knowles T. P. (2016). Kinetic analysis
reveals the diversity of microscopic mechanisms through which molecular
chaperones suppress amyloid formation. Nat.
Commun..

[56] Shammas S. L., Waudby C. A., Wang S., Buell A. K., Knowles T. P., Ecroyd H., Welland M. E., Carver J. A., Dobson C. M., Meehan S. (2011). Binding of the molecular chaperone *α*b-Crystallin to A*β* amyloid
fibrils inhibits
fibril elongation. Biophys. J..

[57] Cohen S. I. A., Arosio P., Presto J., Kurudenkandy F. R., Biverstål H., Dolfe L., Dunning C., Yang X., Frohm B., Vendruscolo M., Johansson J., Dobson C. M., Fisahn A., Knowles T. P., Linse S. (2015). A molecular
chaperone breaks the catalytic cycle that generates toxic A*β* oligomers. Nat. Struct. Mol.
Biol..

[58] Tuttle M.
D., Comellas G., Nieuwkoop A. J. (2016). Solid-state NMR structure
of a pathogenic fibril of full-length human *α*-synuclein. Nat. Struct. Mol. Biol..

[59] Studier F. W. (2005). Protein
production by auto-induction in high density shaking cultures. Protein Expression Purif..

[60] Linse S. (2022). High-Efficiency
Expression and Purification of DNAJB6b Based on the pH-Modulation
of Solubility and Denaturant-Modulation of Size. Molecules.

[61] Hwang T., Shaka A. (1995). Water Suppression That Works. Excitation
Sculpting Using Arbitrary
Wave-Forms and Pulsed-Field Gradients. J. Magn.
Reson., Ser. A.

[62] Adams R. W., Holroyd C. M., Aguilar J. A., Nilsson M., Morris G. A. (2013). “Perfecting”
WATERGATE: Clean proton NMR spectra from aqueous solution. Chem. Commun..

[63] Palmer A. G., Cavanagh J., Wright P. E., Rance M. (1991). Sensitivity improvement
in proton-detected two-dimensional heteronuclear correlation NMR spectroscopy. J. Magn. Reson..

[64] Axell E., Hu J., Lindberg M., Dear A. J., Ortigosa-Pascual L., Andrzejewska E. A., Šneideriene G., Thacker D., Knowles T. P., Sparr E., Linse S. (2024). The role of
shear forces in primary
and secondary nucleation of amyloid fibrils. Proc. Natl. Acad. Sci. U.S.A..

[65] Nozaki Y., Tanford C. (1967). Examination of titration
behavior. Methods Enzymol..

[66] Grey M., Dunning C. J., Gaspar R., Grey C., Brundin P., Sparr E., Linse S. (2015). Acceleration
of *α*-synuclein aggregation by exosomes. J. Biol.
Chem..

[67] Linse S., Sormanni P., O’Connell D. J. (2022). An aggregation inhibitor specific
to oligomeric intermediates of A*β*42 derived
from phage display libraries of stable, small proteins. Proc. Natl. Acad. Sci. U.S.A..

[68] Chia S., Faidon Brotzakis Z., Horne R. I., Possenti A., Mannini B., Cataldi R., Nowinska M., Staats R., Linse S., Knowles T. P., Habchi J., Vendruscolo M. (2023). Structure-Based
Discovery of Small-Molecule Inhibitors of the Autocatalytic Proliferation
of *α*-Synuclein Aggregates. Mol. Pharmaceutics.

[69] Habchi J., Chia S., Limbocker R., Mannini B., Ahn M., Perni M., Hansson O., Arosio P., Kumita J. R., Challa P. K., Cohen S. I., Linse S., Dobson C. M., Knowles T. P., Vendruscolo M. (2017). Systematic
development of small molecules
to inhibit specific microscopic steps of A*β*42 aggregation in Alzheimer’s disease. Proc. Natl. Acad. Sci. U.S.A..

[70] Törner R., Kupreichyk T., Gremer L., Debled E. C., Fenel D., Schemmert S., Gans P., Willbold D., Schoehn G., Hoyer W., Boisbouvier J. (2022). Structural basis for the inhibition
of IAPP fibril formation by the co-chaperonin prefoldin. Nat. Commun..

[71] Gaspar R., Garting T., Stradner A. (2020). Eye lens Crystallin
proteins inhibit
the autocatalytic amyloid amplification nature of mature *α*-synuclein fibrils. PLoS One.

[72] Pálmadóttir T., Waudby C. A., Bernfur K., Christodoulou J., Linse S., Malmendal A. (2023). Morphology-Dependent
Interactions
between *α*-Synuclein Monomers and Fibrils. Int. J Mol. Sci..

[73] Heesink G., van den Oetelaar M. C., Semerdzhiev S. A., Ottmann C., Brunsveld L., Blum C., Claessens M. M. (2024). 14–3-3*τ* as a Modulator of Early *α*-Synuclein Multimerization
and Amyloid Formation. ACS Chem. Neurosci..

[74] Schartman R. R. (2009). On the
thermodynamics of cocrystal formation. Int.
J. Pharm..

[75] Zhang S., Rasmuson C. (2013). Thermodynamics and crystallization of the theophylline-glutaric
acid cocrystal. Cryst. Growth Des..

