# Supplementary material for: The Role of α‑Synuclein–DNAJB6b Coaggregation in Amyloid Suppression
Source: ACS Chem Neurosci. 2025 Apr 30;16(10):1883–97. doi: 10.1021/acschemneuro.4c00883 (PMC12100659; doi:10.1021/acschemneuro.4c00883)
Supplement: Supplementary file 1 [file cn4c00883_si_001.pdf]

*Supporting information*

# The Role of α-Synuclein-DNAJB6b Coaggregation in Amyloid Suppression

Tinna Pálmadóttir[1a*], Josef Getachew[1a], Dev Thacker[1], Johan Wallerstein[2], Ulf Olsson[3], Cecilia Emanuelsson[1], Sara Linse[1*].

[a.] *These authors contributed equally*

[1.] *Biochemistry and Structural Biology, Lund University, Sweden.*

[2.] *Biophysical Chemistry, Lund University, Sweden.*

[3.] *Physical Chemistry, Lund University, Lund, Sweden.*

[*]To whom correspondence should be addressed:
Sara Linse., E-mail: sara.linse@biochemistry.lu.se
Tinna Pálmadóttir., E-mail: tinna.palmadottir@biochemistry.lu.se

## S1. The DNA and protein sequence of wild-type α-syn

*DNA sequence of α-synuclein*

5'ATGGACGTTTTCATGAAAGGTCTGTCTAAAGCTAAAGAAGGTGTTGTTGCTGCTGCTGAAAAAACCA
AACAGGGTGTTGCTGAAGCTGCTGGTAAAACCAAAGAAGGTGTTCTGTACGTTGGTTCTAAAACCAAAG
AAGGTGTTGTTCACGGTGTTGCTACCGTTGCTGAAAAAACCAAAGAACAGGTTACCAACGTTGGTGGTG
CTGTTGTTACCGGTGTTACCGCTGTTGCTCAGAAAACCGTTGAAGGTGCTGGTTCTATCGCTGCTGCTA
CCGGTTTCGTTAAAAAAGACCAGCTGGGTAAAAACGAAGAAGGTGCTCCGCAGGAAGGTATCCTGGAAG
ACATGCCGGTTGACCCGGACAACGAAGCTTACGAAATGCCGTCTGAAGAAGGTTACCAGGACTACGAAC
CGGAAGCTTAATAG

*Protein sequence of human α-synuclein*

MDVFMKGLSKAKEGVVAAAEKTKQGVAEAAGKTKEGVLYVGSKTKEGVVHGVATVAEKTKEQVTNVGGA
VVTGVTAVAQKTVEGAGSIAAATGFVKKDQLGKNEEGAPQEGILEDMPVDPDNEAYEMPSEEGYQDYEP
EA

## S2. Verification of the expression of α-syn

Expression of α-syn in *E.coli* was tested. Samples were prepared according to section 2.1 (main text) and run on SDS-PAGE (see Figure S1). Figure S1 shows that expression was successful. From Figure S1 it can also been seen that significantly lower amounts of α-syn were found within inclusion bodies (solubilized in 8 M urea) and therefore the inclusion bodies were discarded and only the supernatant used for the purification.

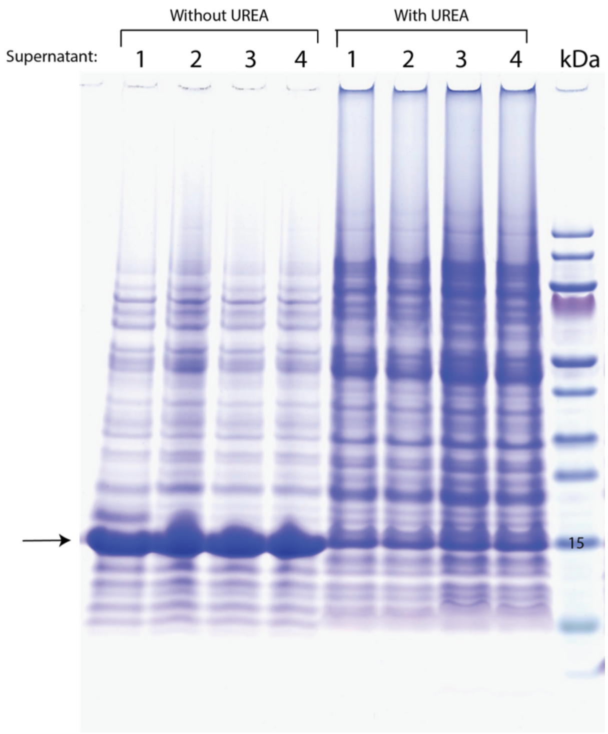


**Figure S1: Verification of the expression of α-syn in *E.coli*.** α-syn was expressed according to section 2.1. Four samples taken from four different baffled flasks were taken at the end of the expression. All samples were analyzed and compared. The arrow points towards the band corresponding to the molecular weight of α-syn.

**S3. Purification of α-syn**

α-syn was purified as described in section 2.2. Here we show an example of analysis after the first IEX purification step (diethylaminoethyl (DEAE) cellulose). An agarose gel electrophoresis was used to determine which fractions contained α-synuclein (Figure S2). In this case fractions 60-72 contained protein corresponding to the molecular weight of pure α-syn that was used as a reference (marked C on the gel). Fractions 58-72 were pooled, diluted in buffer A (1:1) and further purified by DEAE sephacel column

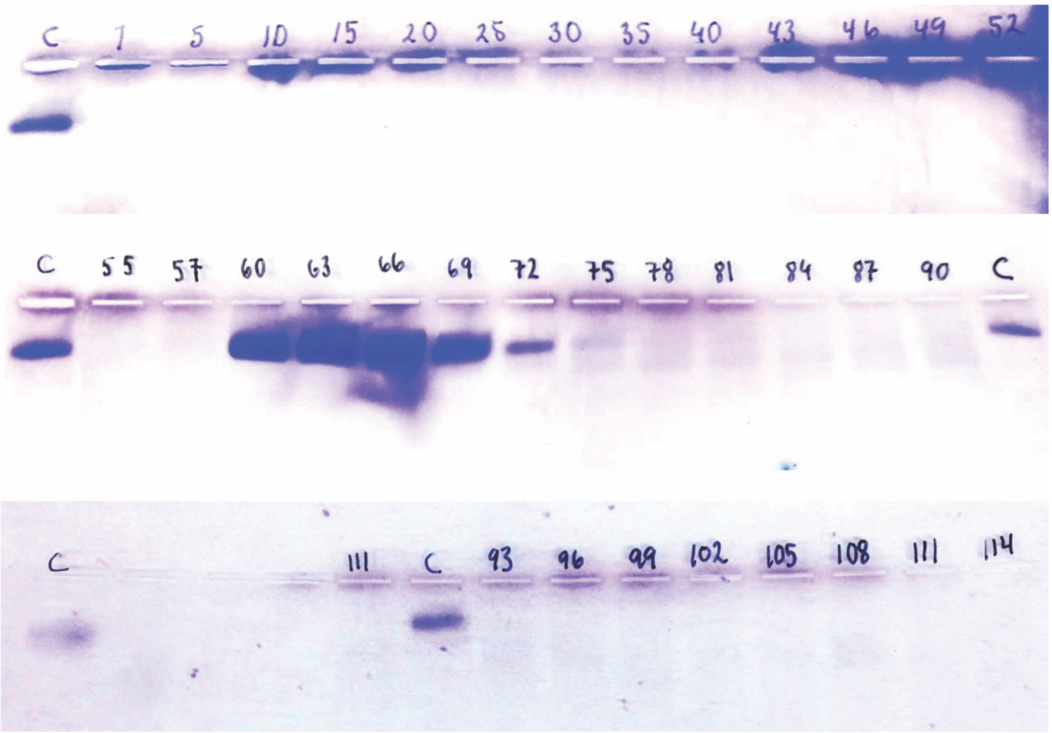


**Figure S2: Fractions collected after first ion-exchange chromatography step (using diethylaminoethyl (DEAE) cellulose) examined using agarose gel electrophoresis.**

The different fractions obtained after the second ion-exchange step were examined by measuring the absorbance at 280 nm in every other collecting tube (Figure S3A). The fractions that showed absorbance at 280 (fractions 47-68) were further analyzed by agarose gel electrophoresis (Figure S3B). In the case shown here, fractions 51-64 contained α-synuclein. Fractions 54-63 were pooled and examined by mass spectrometry and SDS-PAGE for further validation of purity. The SDS-PAGE shows only one protein band, corresponding to α-syn (14.5kDa) (Figure S3C).

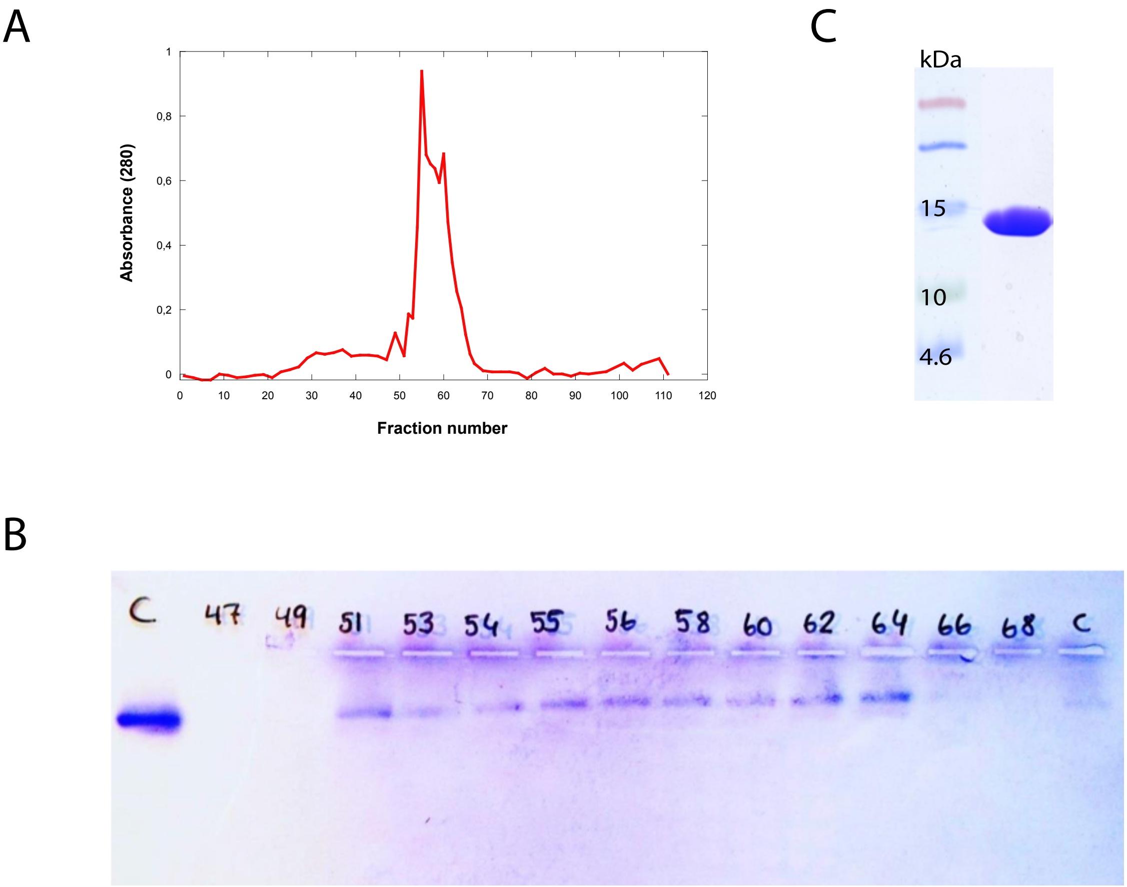


**Figure S3: Fraction obtained after second ion-exchange chromatography step (using diethylaminoethyl DEAE sephacel) examined by A280, agarose gel electrophoresis and SDS-PAGE**. A) The absorbance at 280 nm was measured in every other collecting tube, using NanoDrop. B) Fractions 47-68 were further analyzed by agarose gel electrophoresis. The control sample, C, is previously purified α-synuclein. C) Fractions 54-63 were pooled together and analyzed by SDS-PAGE.

## S4. The DNA and protein sequence of DNAJB6b (JB6)

*DNA sequence of JB6*

5'CATATGGTTGACTACTACGAAGTTCTGGGTGTTCAGCGTCACGCTTCTCCGGAAGACATCAAAAAA
GCTTACCGTAAACTGGCTCTGAAATGGCACCCGGACAAAAACCCGGAAAACAAAGAAGAAGCTGAACG
TAAATTCAAACAGGTTGCTGAAGCTTACGAAGTTCTGTCTGACGCTAAAAAACGTGACATCTACGACA
AATACGGTAAAGAAGGTCTGAACGGTGGTGGTGGTGGTGGTTCTCACTTCGACTCTCCGTTCGAATTC
GGTTTCACCTTCCGTAACCCGGACGACGTTTTCCGTGAATTCTTCGGTGGTCGTGACCCGTTCTCTTT
CGACTTCTTCGAAGACCCGTTCGAAGACTTCTTCGGTAACCGTCGTGGTCCGCGTGGTTCTCGTTCTC
GTGGTACCGGTTCTTTCTTCTCTGCTTTCTCTGGTTTCCCGTCTTTCGGTTCTGGTTTCTCTTCTTTC
GACACCGGTTTCACCTCTTTCGGTTCTCTGGGTCACGGTGGTCTGACCTCTTTCTCTTCTACCTCTTT
CGGTGGTTCTGGTATGGGTAACTTCAAATCTATCTCTACCTCTACCAAAATGGTTAACGGTCGTAAAA
TCACCACCAAACGTATCGTTGAAAACGGTCAGGAACGTGTTGAAGTTGAAGAAGACGGTCAGCTGAAA
TCTCTGACCATCAACGGTAAAGAACAGCTGCTGCGTCTGGACAACAAATAGTAAGGATCC

*Protein sequence of human JB6*

MVDYYEVLGVQRHASPEDIKKAYRKLALKWHPDKNPENKEEAERKFKQVAEAYEVLSDAKKRDIYDKY
GKEGLNGGGGGGSHFDSPFEFGFTFRNPDDVFREFFGGRDPFSFDFFEDPFEDFFGNRRGPRGSRSRG
TGSFFSAFSGFPSFGSGFSSFDTGFTSFGSLGHGGLTSFSSTSFGGSGMGNFKSISTSTKMVNGRKIT
TKRIVENGQERVEVEEDGQLKSLTINGKEQLLRLDNK

**S5. Purity of DNAJB6b batches used in this study.**

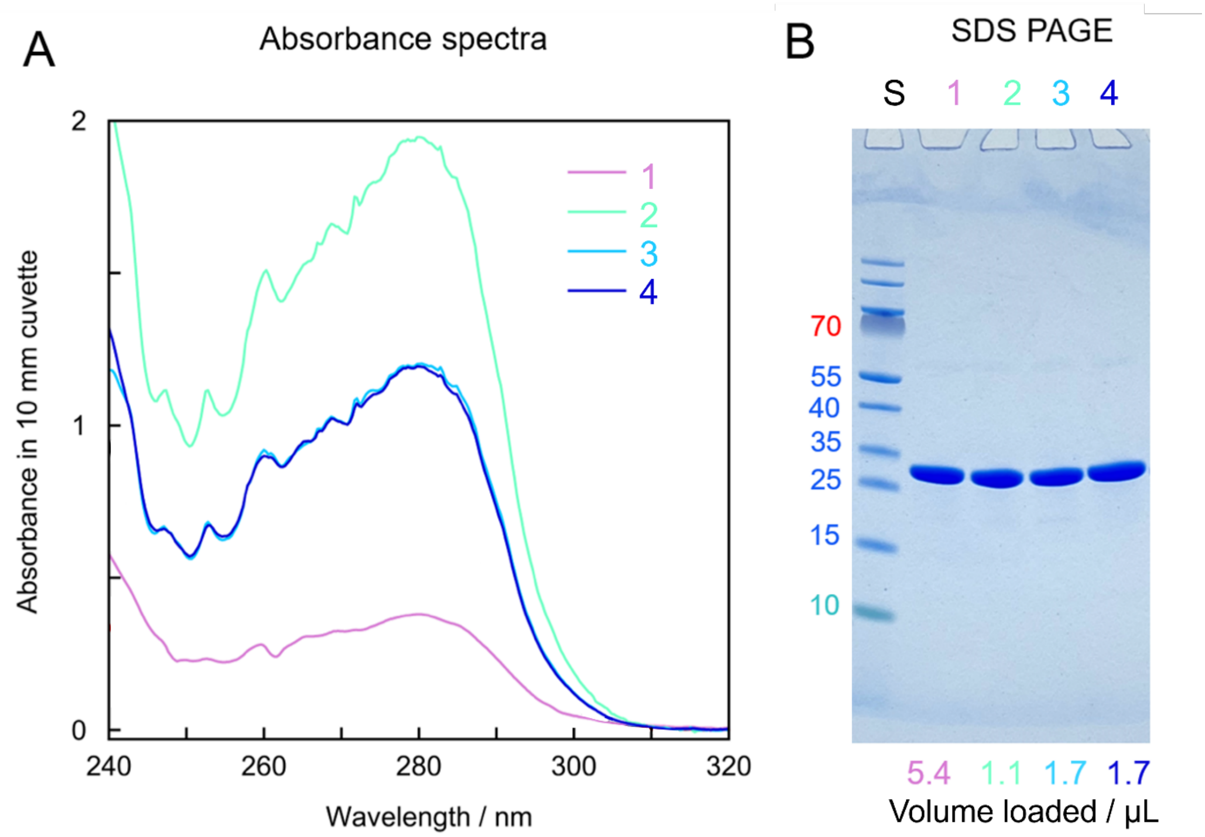


**Figure S4: Comparison of DNAJB6b batches used in this study. A**) Absorbance spectra recorded in a 10 mm quartz cuvette using a Labbot instrument (Probation Labs, Lund, Sweden). **B**) SDS PAGE run on a Novex 10-20% Tris/tricine gel. The volume loaded from each batch is given below the gel. The volumes were calculated based on the absorbance at 280 nm to give the same amount of JB6 per lane (3.8 µg). The band appearing between 55 and 70 is identified by MS as DNAJB6b dimer, which sometimes appears on SDS PAGE.

Batch numbering in panels A and B of Figure S4:

1. JB6 wt used in the cryo-TEM experiments at pH 5.5 (Supplementary information Figure S17).
2. JB6 wt used in the cryo-TEM experiments at pH 4.5 (Figure 6).
3. JB6 wt used in the inhibition and solubility experiments for quantification by HPLC (Figure 2 and 3).
4. JB6 wt used in the inhibition and solubility experiments by NMR spectroscopy (Figure 4).

All batches were purified in-house according to the protocol by Linse 2022 (Ref 60). We conclude that the JB6 is highly pure and contain maximum 0.3-1.0% impurities.

## S6. Size exclusion and kinetic analysis

Before each experiment, monomeric α-syn was freshly prepared using size exclusion chromatography (Figure S5A). The center of the monomeric peak was collected and used for kinetic experiments (Figure S5B). The quality and purity of the monomeric sample was also analyzed by mass spectrometry.

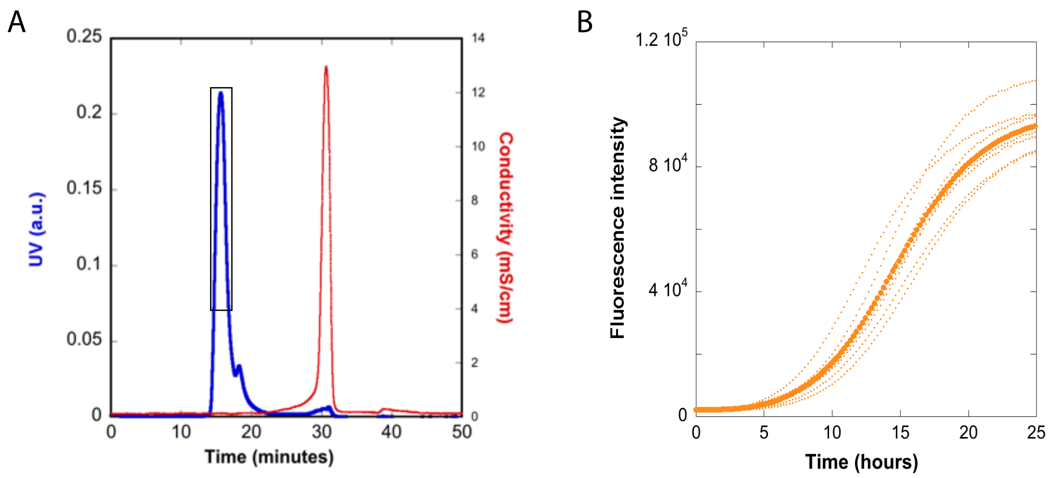


**Figure S5: An example of SEC chromatogram and ThT-kinetics of purified sample**. A) This data was obtained during preparation of α-syn for experiments in 10 mM Mes, 0.02% $NaN_3$, pH 5.5. Center of the monomeric peak (highlighted by a black box) is collected and used for the kinetics experiments. B) Kinetic experiments of nine replicates, and the average is shown in bold. 20 uM α-syn with 1% seeds in 10 mM Mes pH 5.5.

**S7. Solubility measurements of α-syn with JB6 at pH 5.5**

Aggregation kinetics of 20 µM α-syn with different concentrations JB6, at pH 5.5 is shown in Figure S6. Three replicates, of each condition, were measured from the beginning of the experiment. For monomer quantification by using HPLC-MS and SDS-PAGE, one replicate from each condition (different α-syn and JB6 ratios) was collected at three different time points (45 h, 140 h and 188h), samples were spun down and supernatant analyzed (described in section 2.6.2, main text). Figure S6 shows the full kinetics over the 188 h, where ThT plateau is reached for all JB6 concentrations except for 125 nM.

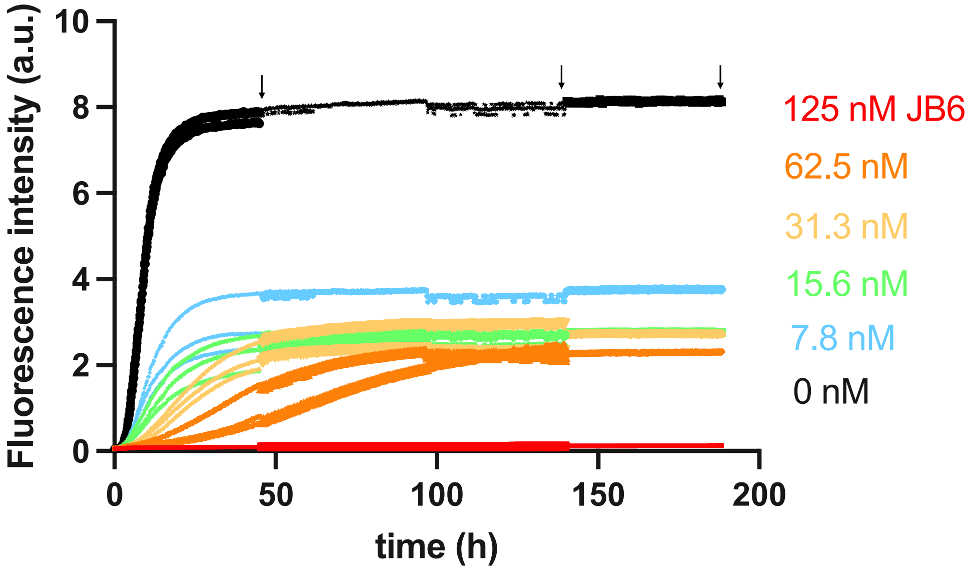


**Figure S6: Kinetics of 20 µM α-syn with different concentrations JB6, at pH 5.5 in 10 mM MES, 1.4 mM NaP and 0.02% $NaN_3$ at 37°C.** Three replicates are shown for each JB6 concentration. After 45 h, 140 h and 188h (arrows) one replicate from each JB6 concentration was collected and further analyzed with HPLC-MS and SDS-PAGE.

Figure S7 shows the HPLC-MS traces of 20 µM α-syn with different concentrations of JB6, after 45 h incubation. The peak observed after 1 min corresponds to the buffer. The peak appearing shortly before 4 min only appeared in samples incubated in PEGylated plates and is, therefore, corresponding to PEG. Shortly after 4 min, α-syn eluted. The full chromatogram is 15 minutes, however there are no visible traces beyond the eluted α-syn, thus Figure S7 shows the HPLC-MS traces only up to 6 min.

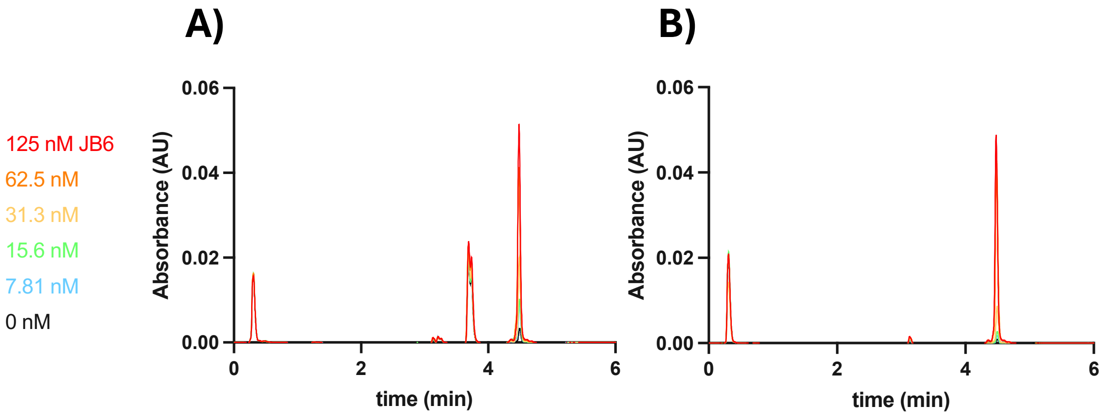


**Figure S7: HPLC-MS traces of supernatants collected after 45h incubation of 20 µM α-syn in the presence of different concentrations of JB6.** The HPLC-MS traces are shown from 1 to 6 min, where absorbance was measured at 280 nm. **A)** Samples were withdrawn from the kinetic experiment presented in Figure S6. The peak at ~3.8 min corresponds to PEG. **B)** An example of HPLC-MS traces of samples, not incubated in PEGylated plates, showing no detectable peak at ~3.8 min.

Figure S8 show an SDS-PAGE of supernatants from the samples collected after 140 h and 188 h (corresponding to the arrows shown Figure S6). The SDS-PAGE of samples analyzed after 45h incubation is shown in the main text. It is evident that there is an increase in monomer concentration for samples with higher amounts of JB6. Furthermore, the intensities stay relatively constant between the two time points, which suggests that equilibrium has been reached. Degradation/truncation of α-syn was detected after 140h and 188h. Truncation at residue Asp119 has been observed earlier after prolonged incubation of α-syn[1,2,3]. No truncation was observed after 45 h incubation (see main text).

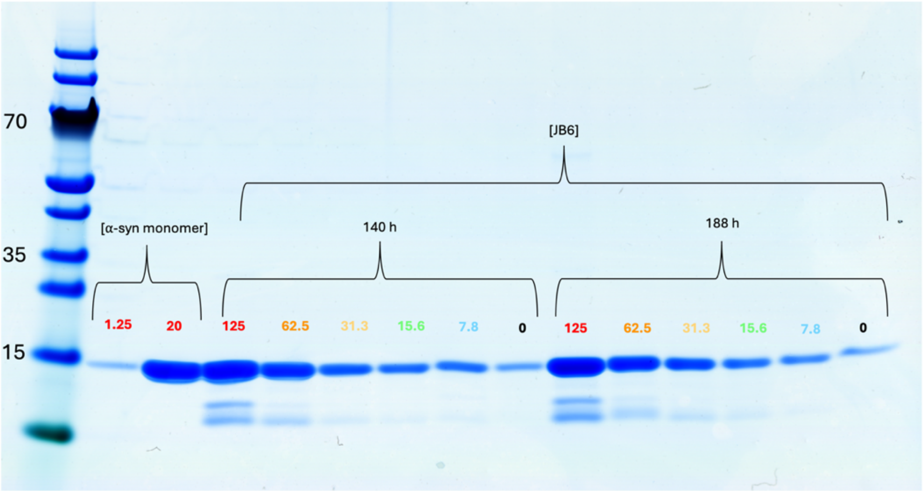


**Figure S8: SDS-PAGE of supernatant from samples in Figure S6 and pure monomeric α-syn.** Samples were collected after 140 h and 188 h incubation and centrifuged, (see, section 2.6.2, main text) from which the supernatant was mixed 1:1 with loading buffer and 10 µl was loaded to the gel. InstantBlue® Coomassie Protein Stain (ISB1L) (Abcam Ltd) was used for staining. The pure monomeric α-syn was used as a concentration reference and was not incubated.

## S8. NMR experiments at low α-syn:JB6 molar ratio

The first NMR experiment was conducted for samples consisting of 20 µM $^{15}$N labeled α-syn, 1% seeds, 2.5 µM free $^{15}$N labeled tryptophan (trp), DSS, 10% $D_2O$, prepared in 10 mM MES, 1.4 mM NaP, 0.02% $NaN_3$, pH 5.5. 550 µl samples were prepared with or without 31.3 nM DNAJB6b. The spectra were recorded using a Bruker Avance Neo 600 MHz (Bruker Biospin, Rheinstetten, Germany) with a QCI(P) 5mm CryoProbe. The spectra were acquired in close to identical manner as described in section 2.6.3. except for the lower magnetic field strength resulting in spectral width of 16 ppm and an acquisition time of 1.7 s. The interscan delay was set to 1 s. We find that supplementing the samples with 10 µM tryptophan does not have any significant influence on the aggregation kinetics of 20 µM α-syn in absence and presence of JB6 (see Figure S9).

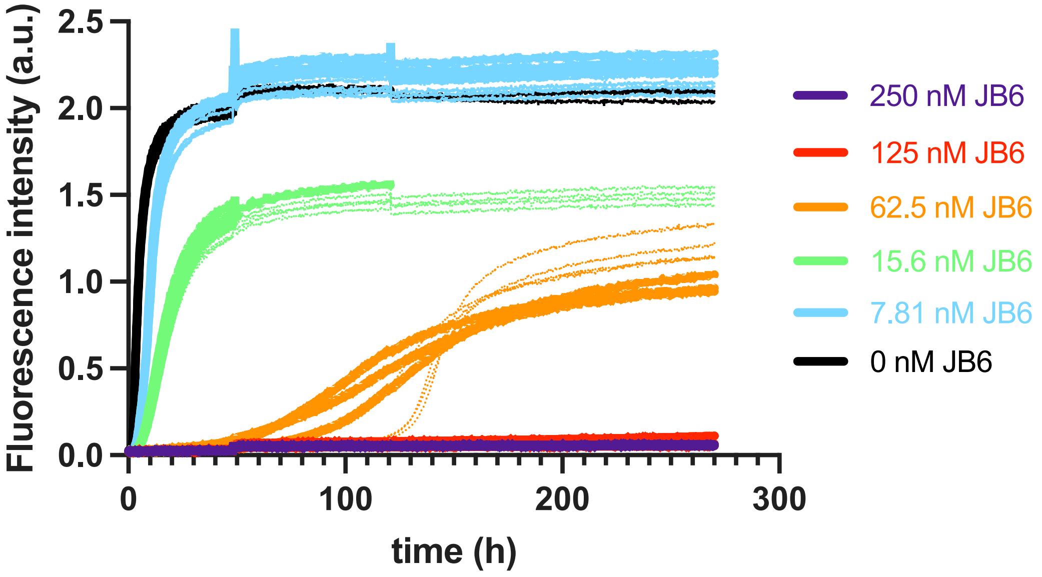


**Figure S9: ThT fluorescence kinetics of α-syn in presence of JB6 w/wo 10 µM Trp.** Data points with Trp are plotted with dashed markers and without Trp are with thick lines.

The decrease in detected monomer concentration as seen in Figure S10 shows no detectable inhibition with the addition of 31.3 nM JB6 to 20 µM α-syn.

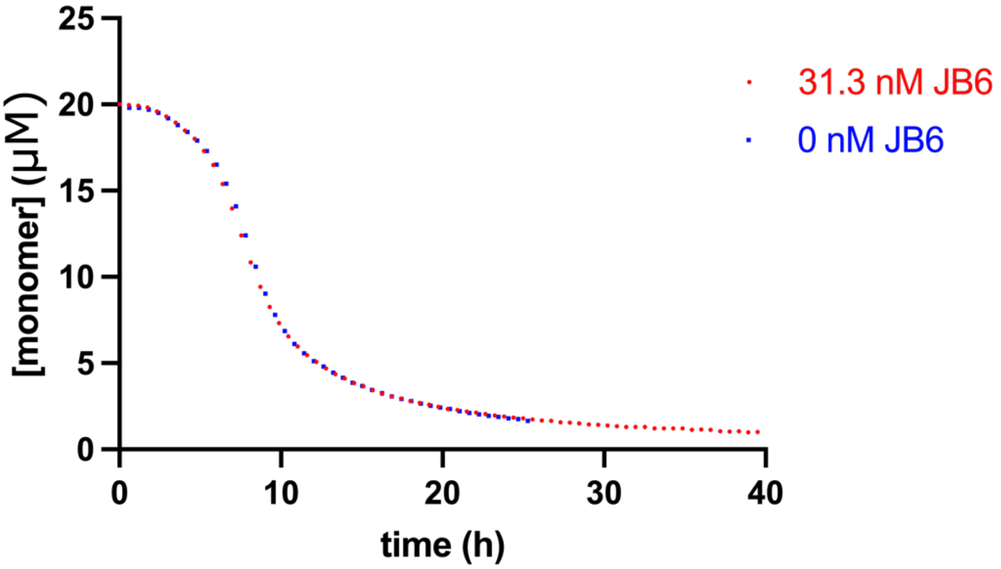


**Figure S10: 1D $^{1}$H NMR measurements of α-syn with or without 31.3 nM JB6 at pH 5.5 in 10 mM MES, 1.4 mM NaP and 0.02% $NaN_3$ at 37°C.** Observed α-syn monomer concentration, determined through peak integration of the complete amide region in 1D $^{1}$H spectra. No inhibition of α-syn aggregation was observed.

The cause of this could be the altering of sample container. Comparing the plates used for HPLC-MS and the NMR tubes, there are differences in surface properties, in surface to volume ratios, which furthermore changes the air-water interface, and agitation[4]. These factors impact the nucleation of α-syn which may alter the necessary molar ratio required to attain detectable inhibition. Furthermore, we show that JB6 is affected by the change in sample containers (NMR tubes vs. low binding plates), where JB6 adsorbs to the glass surface of the NMR tubes (see section S9).

**S9. The adsorption of JB6 to NMR tubes**

The adsorption of JB6 to the surfaces of NMR tubes and low-binding plates were investigated and compared. This was done with the aim of understanding the need of higher concentrations of JB6 in the NMR experiments to obtain comparable retardation as was observed in the kinetic experiments performed in low-binding plates. The adsorption to these surfaces was measured by HPLC-MS and SDS-PAGE, according to section 2.11. As seen by HPLC-MS (Figure S11A and B), there is significant adsorption of JB6 (sample concentration of 250 nM) to the NMR tubes already after 1.5 hour, while no adsorption is detected for the plates. This is supported by the SDS-PAGE analysis, showing significant depletion of JB6 from solution after 1.5 hour incubation in the NMR tubes while no significant effect is observed in case of the low binding plates. By SDS-PAGE analysis (Figure S11D and E), we detect no JB6 in solution after 4 hours and 1 day in the NMR tubes in the samples of 250 nM JB6, while its concentration remains unchanged from $t_0$ in the case of the low-binding plates. The samples consisting of 2 μM JB6 are less affected by the adsorption to the surface of the NMR tubes (Figure S11F).

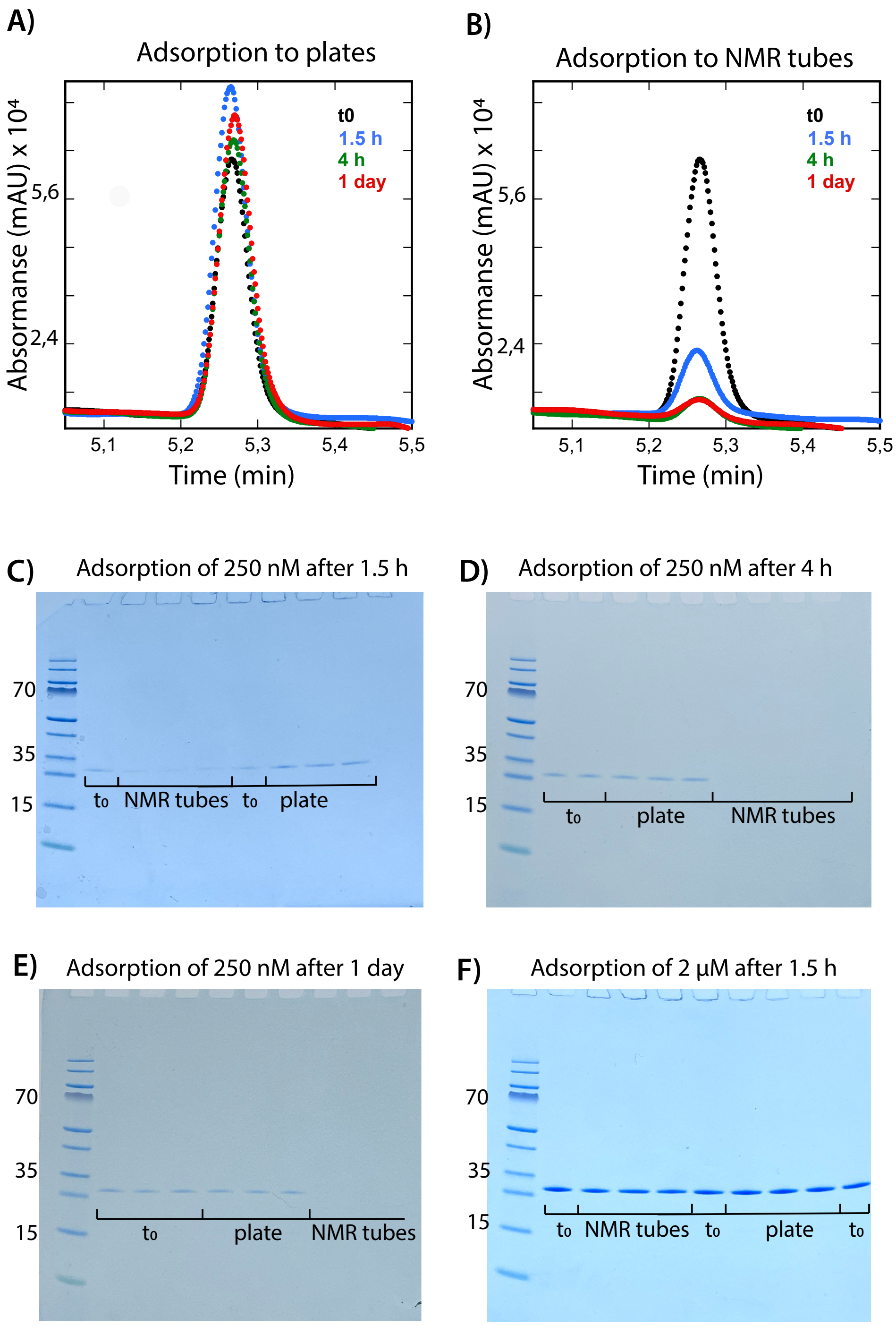


**Figure S11: Adsorption of JB6 to NMR tubes and low-binding plates at pH 5.5.** A) Adsorption of 250 nM JB6 to low-binding plates, measured with absorbance at 205 nm on HPLC-MS. B) Adsorption of 250 nM JB6 to NMR tubes, measured with absorbance at 205 nm on HPLC-MS. C) Adsorption of 250 nM JB6 after 1.5 hour measured by SDS-PAGE. 15 µL of sample added to each well. D) Adsorption of 250 nM JB6 after 4 h measured by SDS-PAGE. 20 µL of sample added to each well. E) Adsorption of 250 nM JB6 after 1 day measured by SDS-PAGE. 20 µL of sample added to each well. F) Adsorption of 2 µM JB6 to NMR tubes and low binding plates after 1.5 h hours measured by SDS-PAGE, 15 µL of sample added to each well. 3 µL of ladder was loaded in all cases (C-F).

**S10. Effect of JB6 on residue resolved signals of free α-syn monomer measured by NMR spectroscopy.**

As a complement to Figure 4B, superimposed $^{1}H$-$^{15}N$ HSQC spectra at the respective $t_{1/2}$ of the kinetics in Figure 4A is shown in Figure S12A. There are no significant differences in the chemical shifts of the free α-syn monomer at start of the experiment ($t_0$) and the respective $t_{1/2}$. Furthermore, we do not detect chemical shifts in superimposed $^{1}H$-$^{15}N$ HSQC spectrum of α-syn incubated for 2 days with 2 μM JB6, over the initial α-syn alone spectrum (see Figure S12B).

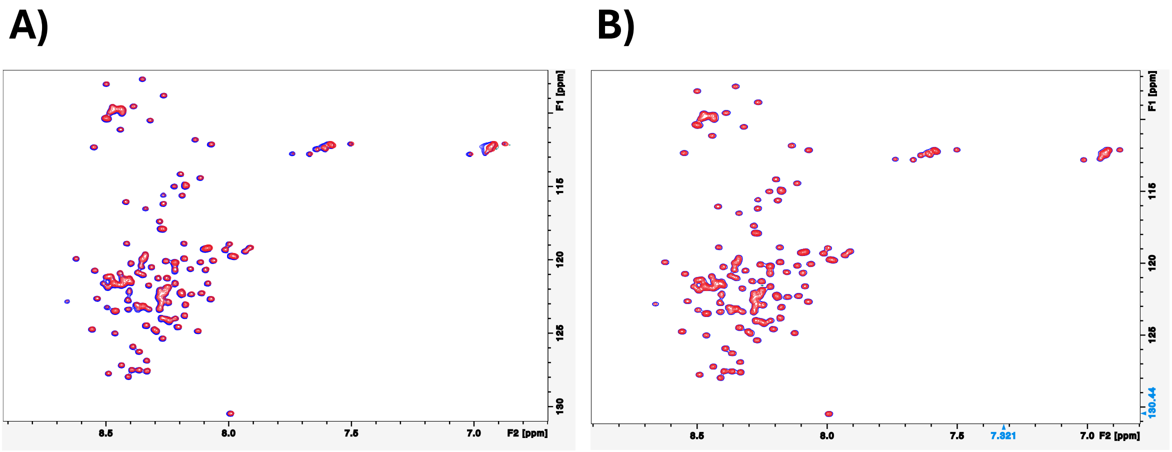


**Figure S12: Superimposed $^{1}H$-$^{15}N$ HSQC spectra on α-syn with and without JB6 at pH 5.5, 37°C**. A) Recorded at the respective $t_{1/2}$ of α-syn with 250 nM JB6 in red and without JB6 in blue. B) Recorded after 2 days incubation of α-syn with 2 μM JB6 in red and initial spectrum without JB6 in blue. F1 on the y-axis represents $^{15}N$ signals and F2 on the x-axis the $^{1}H$ signals. The figure was produced in TopSpin 4.3.0.

**S11. Cryo-TEM of α-syn in the presence of JB6 at pH 4.5**

Aggregation of 20 μM α-syn in the presence of 2 μM (10%) or 125 nM (0.6%) JB6 was followed at pH 4.5 in 10 mM acetate buffer, 0.02% $NaN_3$ by monitoring the change in ThT fluorescence with time. The aggregation was compared to a sample of α-syn alone and JB6 alone (see main text and Figure 6). Samples were collected at the end of the aggregation experiment (after 116 h, see Figure 6) and then analyzed with cryo-TEM. The ultrastructure of α-syn was found to be remarkably different in the presence of JB6 indicating the formation of coaggregates of α-syn and JB6 (see main text and Figure 6). In Figures S13 to S16 we show additional images of the same samples as presented in Figure 6.

nm.

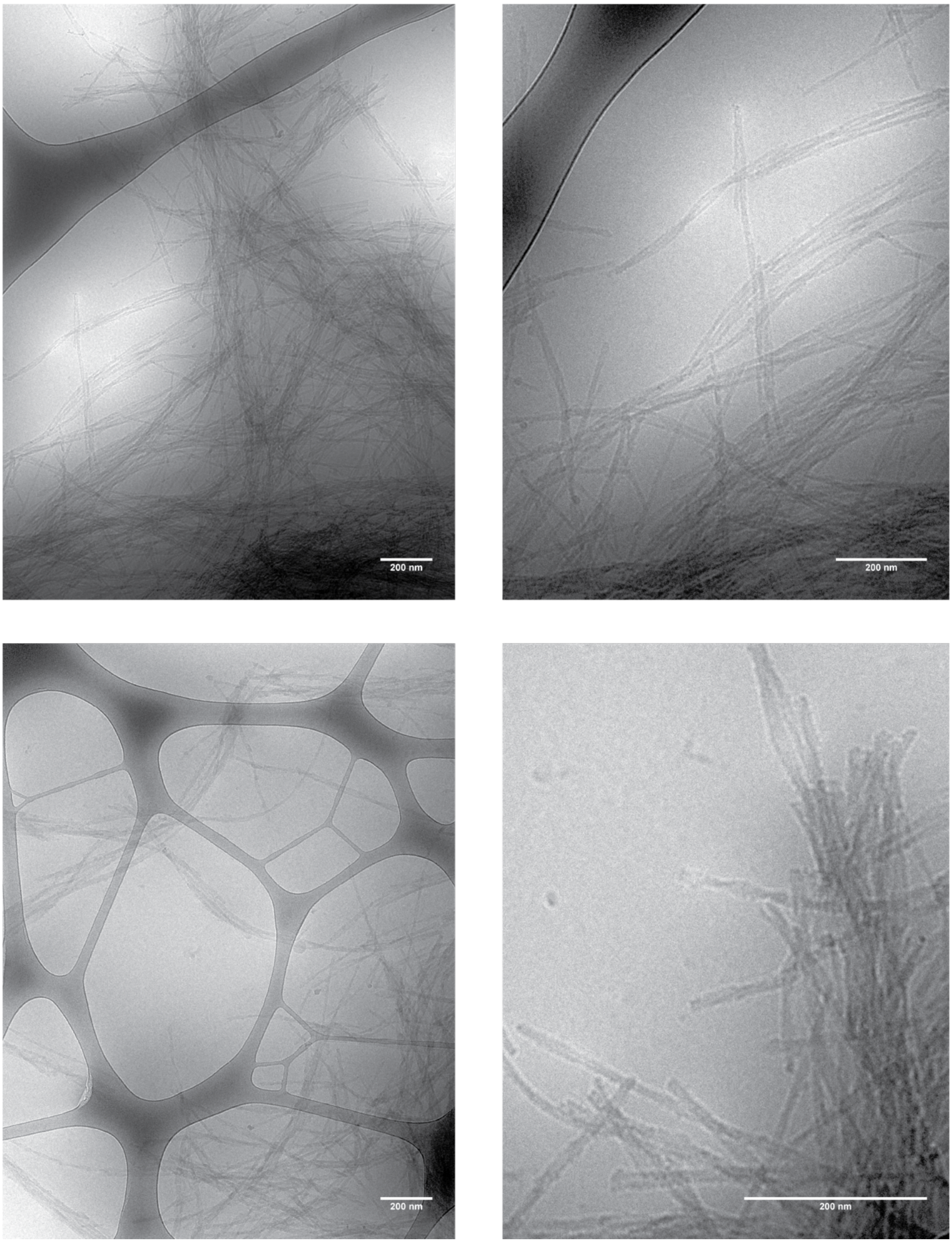


**Figure S13: Cryo-TEM images of 20 µM α-syn alone at pH 4.5 (10 mM acetate buffer, 0.02% NaN3).** Cryo-TEM images showing different ultrastructure between the samples collected at the end of a kinetic experiment (after 116 h), when ThT fluorescence had reached the final plateau (see Figure 6, main text). The scale bars correspond to 200 nm.

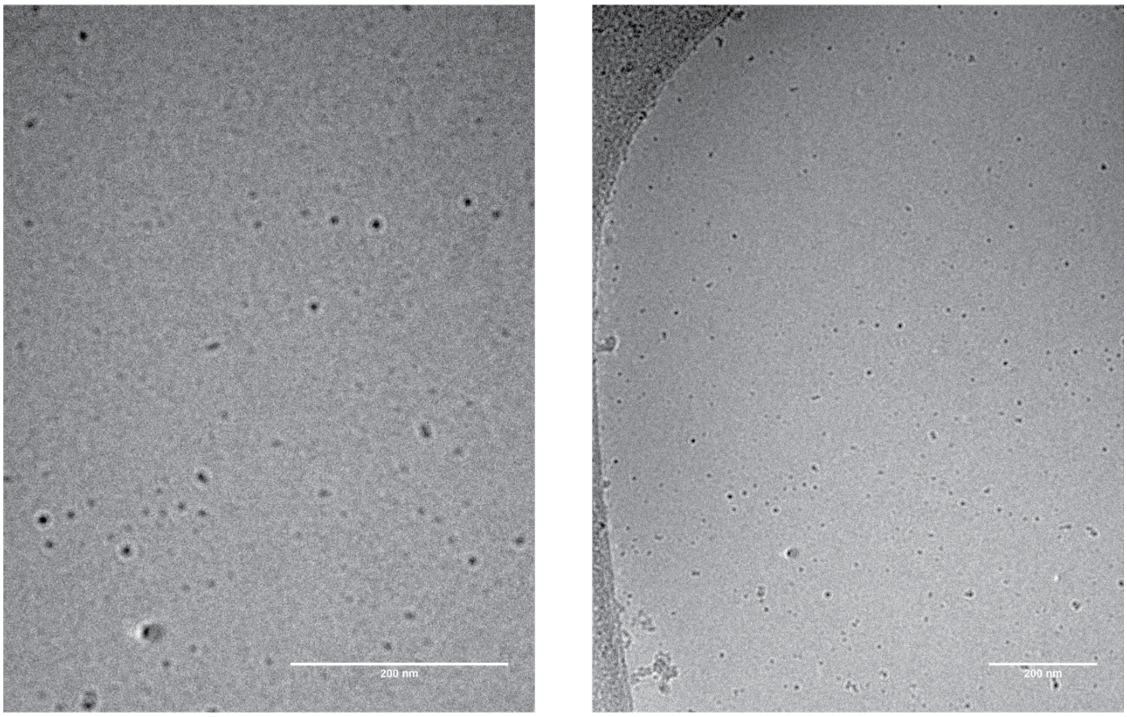


**Figure S14: Cryo-TEM images of 2 µM JB6 alone at pH 4.5 (10 mM acetate buffer, 0.02% $NaN_3$).** Cryo-TEM images of JB6 alone, after incubation at 37°C for the same time and at the same conditions as samples shown in Figures S13, S15-S16. Samples were collected at the end of the experiment (after 116 h), shown in Figure 6 (main text). The scale bars correspond to 200 nm.

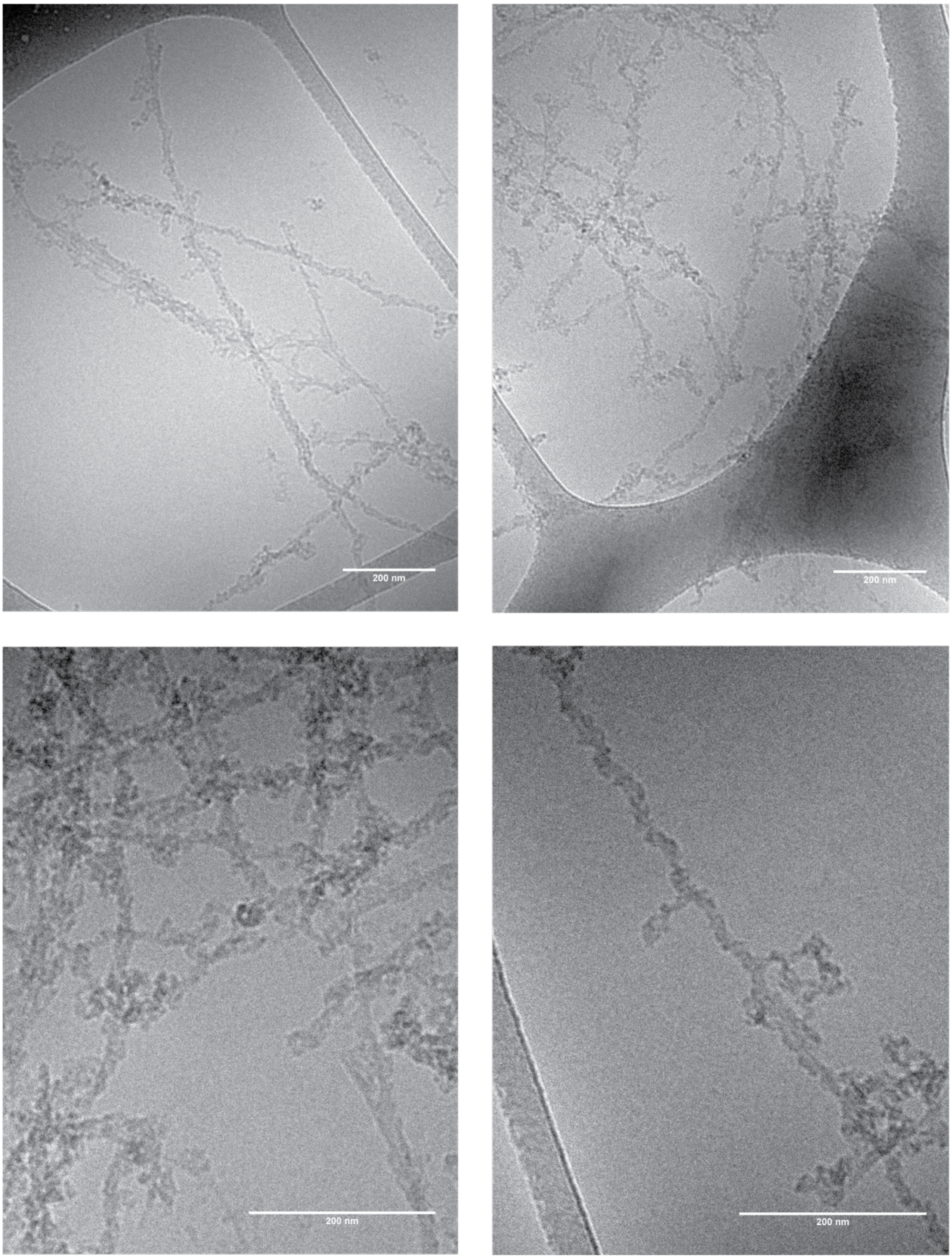


**Figure S15: Cryo-TEM images of 20 µM α-syn in the presence of 2 µM (10%) JB6 at pH 4.5 (10 mM acetate buffer, 0.02% $NaN_3$).** Cryo-TEM images showing different ultrastructure between the samples collected at the end of a kinetic experiment (after 116 h),, when ThT fluorescence had reached the final plateau (see Figure 6, main text). The scale bars correspond to 200 nm.

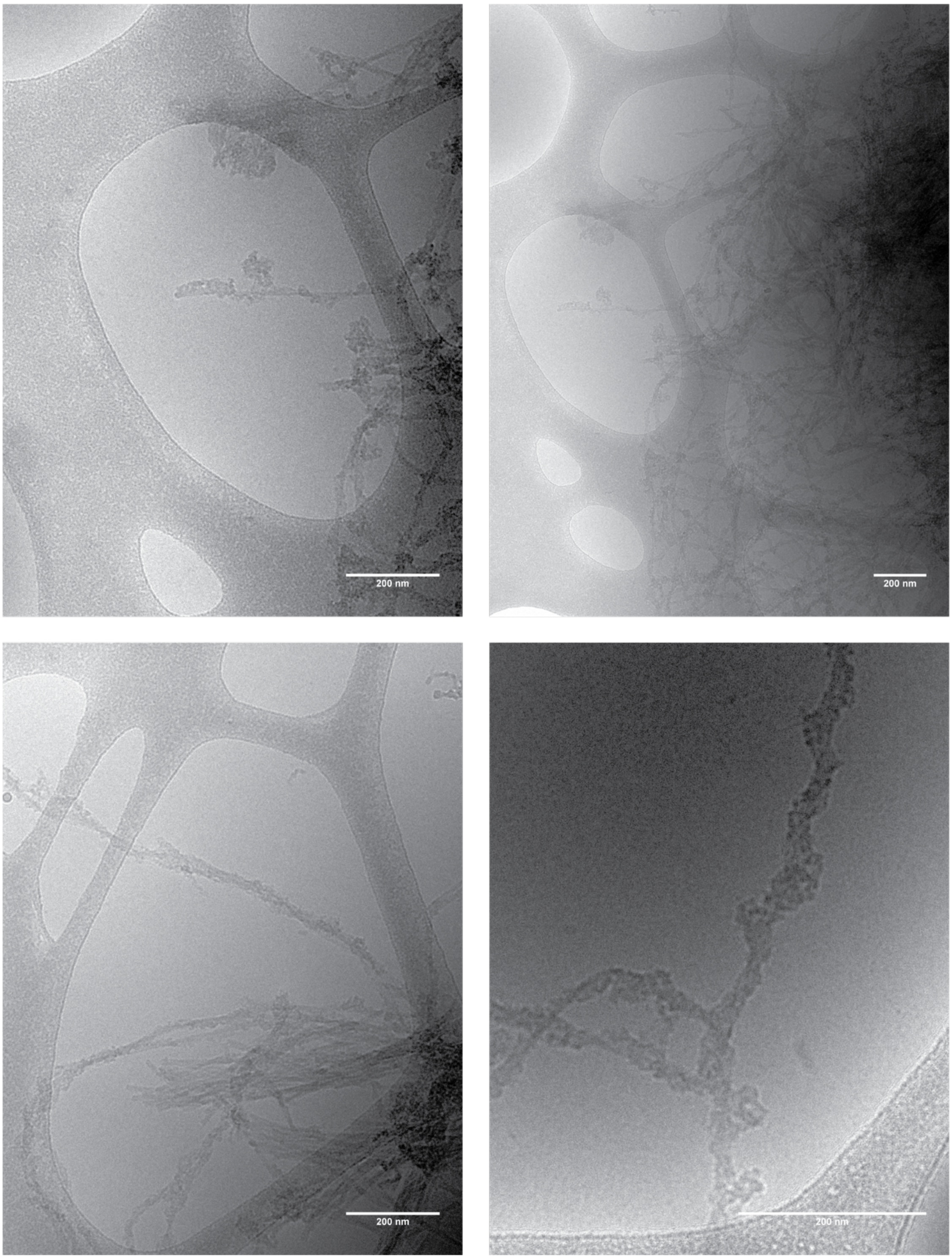


**Figure S16: Cryo-TEM images of 20 µM α-syn in the presence of 125 nM (0.6%) JB6 at pH 4.5 (10 mM acetate buffer, 0.02% $NaN_3$).** Cryo-TEM images showing different ultrastructure between the samples collected at the end of a kinetic experiment (after 116 h), when ThT fluorescence had reached the final plateau (see Figure 6, main text). The scale bars correspond to 200 nm.

**S12. Cryo-TEM and corresponding kinetics at pH 5.5**

The structure of α-syn in the presence of different concentration of JB6 was investigated by cryo-TEM at pH 5.5 in 10 mM Mes, 1.4 mM NaPB, 0.02% $NaN_3$. 20 μM α-syn was aggregated in the presence of 0.02, 0.06, 0.2, 0.6 and 2 μM JB6, or 0.1, 0.3, 1.0, 3.0 and 10%, respectively (Figure S17A). The sample were taken at the end of the aggregation experiment shown in Figure S17A and analyzed with cryo-TEM (Figure S17B). The morphology of α-syn was found to change in the presence of JB6 (Figure S17B), with less clear fibrils and shorter aggregates. Aggregates were detected in all samples, including the samples showing no increase in ThT fluorescence intensity. This is consistent with experiments performed at pH 4.5, where aggregates were also detected with cryo-TEM in the samples having little increase in the ThT fluorescence curves, this is also consistent to the results presented in section 3.4.2 on the detection of ThT-negative aggregates.

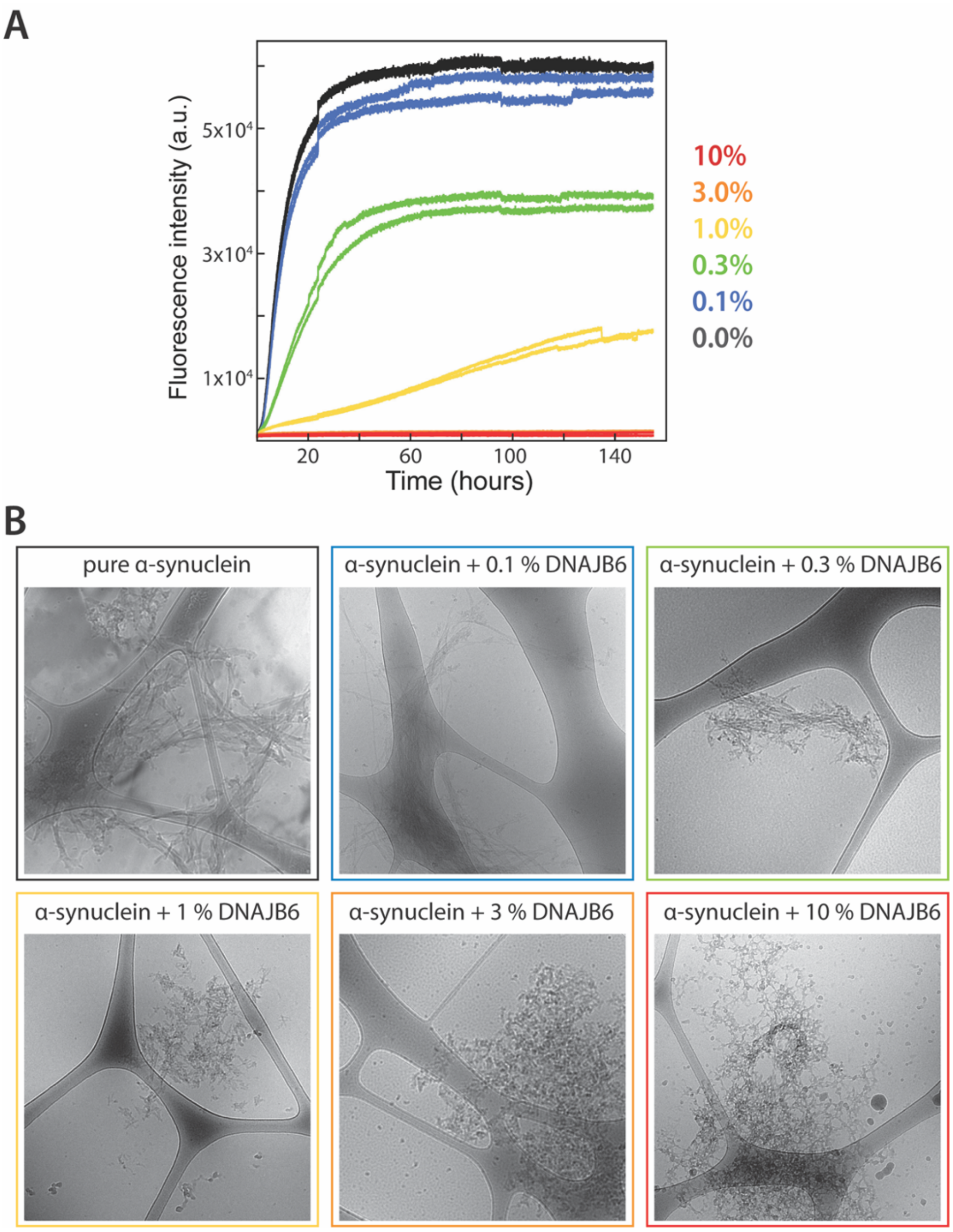


**Figure S17: Kinetics and cryo-TEM analysis of α-syn in the presence of different concentrations of JB6**. Final concentration of α-syn was 20 µM in all samples. Concentrations of JB6 are shown as a percentage of 20 µM. A) Aggregation kinetics at pH 5.5 in, 10 mM Mes, 1.4 mM NaPB, 0.02% $NaN_3$. B) Cryo-TEM of samples taken at the end-point of the aggregation experiment (150 h).

### S13. JB6 in supernatant at pH 4.5

JB6 alone was incubated at pH 4.5 in low-binding tubes at 37°C for two days (Figure S18). SDS-PAGE on the JB6 before and after centrifugation show JB6 in both samples. This indicates the formation of larger structures of JB6, possibly coaggregates with α-syn as it is depleted from the supernatant in presence of α-syn (see Figure 8, main text).

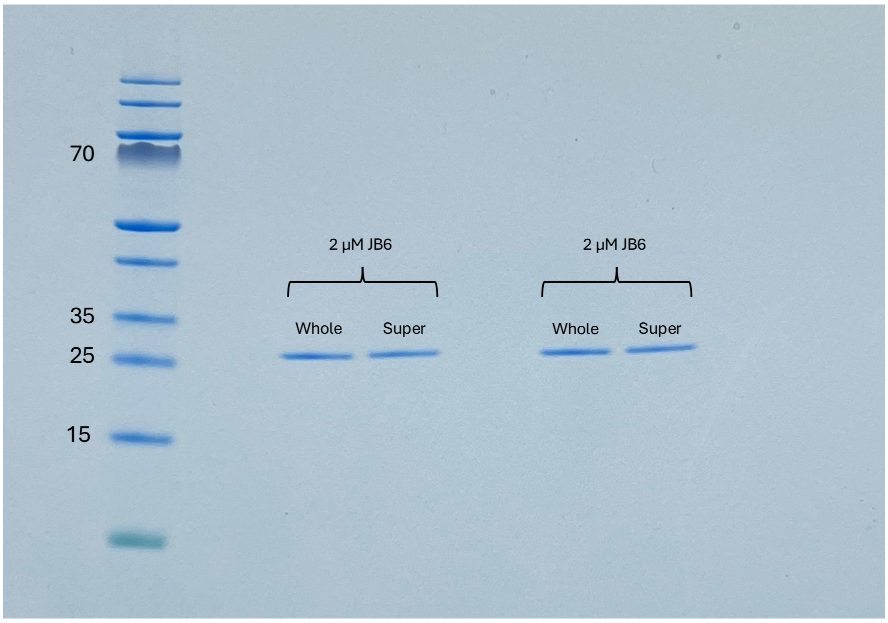


**Figure S18: SDS-PAGE of JB6 at pH 4.5.** Samples of 2 µM JB6 incubated for 2 days at 37°C. Whole samples and supernatant after centrifugation added to SDS-PAGE. JB6 detected in both samples**.**

## References


1. Anderson JP, Walker DE, Goldstein JM, de Laat R, Banducci K, Caccavello RJ, Barbour R, Huang J, Kling K, Lee M, Diep L, Keim PS, Shen X, Chataway T, Schlossmacher MG, Seubert P, Schenk D, Sinha S, Gai WP, Chilcote TJ. Phosphorylation of Ser-129 is the dominant pathological modification of alpha-synuclein in familial and sporadic Lewy body disease. J Biol Chem. 2006;281(40):29739-52.

2. Ariesandi W, Chang CF, Chen TE, Chen YR. Temperature-dependent structural changes of Parkinson's alpha-synuclein reveal the role of pre-existing oligomers in alpha-synuclein fibrillization. PLoS One. 2013;8(1).

3. Ortigosa-Pascual L, Leiding T, Linse S, Pálmadóttir T. Photo-Induced Cross-Linking of Unmodified α-Synuclein Oligomers. ACS Chem Neurosci. 2023;14(17):3192-3205.

4. Axell E, Hu J, Lindberg M, Dear AJ, Ortigosa-Pascual L, Andrzejewske EA, Sneideriené G, Thacker D, Knowles TPJ, Sparr E, Linse S. The role of shear forces in primary and secondary nucleation of amyloid fibrils. Proc Natl Acad Sci USA. 2024; 121(25).
